# Drifting along: using diatoms to track the contribution of microbial mats to particulate organic matter transport in a glacial meltwater stream in the McMurdo Dry Valleys, Antarctica

**DOI:** 10.3389/fmicb.2024.1352666

**Published:** 2024-05-09

**Authors:** Lee F. Stanish, Tyler J. Kohler, Joshua Darling, Diane M. McKnight

**Affiliations:** ^1^Institute of Arctic and Alpine Research, University of Colorado, Boulder, CO, United States; ^2^Department of Ecology, Faculty of Science, Charles University, Prague, Czechia; ^3^Department of Civil and Environmental Engineering, University of Colorado, Boulder, CO, United States

**Keywords:** flow regime, microbial mat, cyanobacteria, *Nostoc*, diatom, hydrology

## Abstract

Flow pulses mobilize particulate organic matter (POM) in streams from the surrounding landscape and streambed. This POM serves as a source of energy and nutrients, as well as a means for organismal dispersal, to downstream communities. In the barren terrestrial landscape of the McMurdo Dry Valleys (MDV) of Antarctica, benthic microbial mats occupying different in-stream habitat types are the dominant POM source in the many glacier-fed streams. Many of these streams experience daily flow peaks that mobilize POM, and diatoms recovered from underlying stream sediments suggest that mat-derived diatoms in the POM are retained there through hyporheic exchange. Yet, ‘how much’ and ‘when’ different in-stream habitat types contribute to POM diatom assemblages is unknown. To quantify the contribution of different in-stream habitat types to POM diatom assemblages, we collected time-integrated POM samples over four diel experiments, which spanned a gradient of flow conditions over three summers. Diatoms from POM samples were identified, quantified, and compared with dominant habitat types (i.e., benthic ‘orange’ mats, marginal ‘black’ mats, and bare sediments). Like bulk POM, diatom cell concentrations followed a clockwise hysteresis pattern with stream discharge over the daily flow cycles, indicating supply limitation. Diatom community analyses showed that different habitat types harbor distinct diatom communities, and mixing models revealed that a substantial proportion of POM diatoms originated from bare sediments during baseflow conditions. Meanwhile, orange and black mats contribute diatoms to POM primarily during daily flow peaks when both cell concentrations and discharge are highest, making mats the most important contributors to POM diatom assemblages at high flows. These observations may help explain the presence of mat-derived diatoms in hyporheic sediments. Our results thus indicate a varying importance of different in-stream habitats to POM generation and export on daily to seasonal timescales, with implications for biogeochemical cycling and the local diatom metacommunity.

## 1 Introduction

The processes controlling the quantity and quality of organic matter (OM) in stream ecosystems are critical for understanding stream ecosystem functioning. For example, knowledge on the source and dynamics of OM can help explain stream resource abundance and quality, and ultimately shed light upon important ecological processes such as decomposition, mineralization, and respiration rates (Tank et al., 2010; Battin et al., 2023). For most headwater streams, OM is viewed as being derived from a combination of allochthonous and autochthonous inputs (Vannote et al., 1980). However, in streams draining the world’s alpine and polar regions, allochthonous inputs (from leaf litter, for example) are scarce, placing a much greater reliance on autochthonous sources such as benthic photoautotrophs (Kohler et al., 2022). Therefore, in streams like these, understanding the in-stream sources of autochthonous OM, and subsequent controls on its transport, is paramount to understanding local biogeochemical processes and nutrient flows. Furthermore, climatic changes in polar and alpine regions are leading to changes in hydrological flow regimes (IPCC, 2019), including an increase in the magnitude and frequency of scouring flood events, that could alter in-stream OM sources and lead to changes in OM quality and quantity over time.

The glacier-fed streams of the McMurdo Dry Valleys (MDV) in Antarctica provide an excellent setting to study how particulate organic matter (POM) is mobilized and transported within streams. Given the barren terrestrial landscape of this polar desert, lateral inputs of allochthonous POM and water are mostly lacking, and POM transported in streams solely originates from autochthonous sources (Cullis et al., 2014). Two distinct, macroscopic vegetation types dominate many MDV streams; orange (or red) microbial mats formed of filamentous oscillatorian cyanobacteria and adhering to sediments within stream channels, and accumulations of black mucilagenous colonies of *Nostoc* weakly attached to sediments along stream margins (Alger et al., 1997; Kohler et al., 2015c). These latter ‘black mats’ are particularly important to local nutrient cycles given their ability to fix nitrogen, and the remineralization of both mat types in the hyporheic zone are critical to balancing downstream nutrient budgets (Kohler et al., 2018, 2023) given high rates of biological uptake (McKnight et al., 2004).

The MDV microbial mats are adapted to harsh hydrological conditions, existing in a freeze-dried state throughout the winter, and resuming growth when water becomes available in summer (McKnight et al., 1999). Given that streamflow is generated solely by glacial melt, discharge is inextricably related to solar radiation. Thus, many MDV streams exhibit daily flow cycles that produce a conspicuous daily flow peak coinciding with the period of the day when the sun shines most directly on the source glacier face (Conovitz et al., 1998). These daily flow cycles in turn create distinct periods of POM generation, mobilization, and transport. For example, Cullis et al. (2014) demonstrated that over a 24-h period, POM in transport exhibits the highest concentrations on the rising limb of the daily flow peak, indicating supply limitation. Yet, the relative contributions of the resident orange versus black mats to the POM over the daily flow cycle remains unresolved, despite the relevance of this question to interpreting long-term relationships between hydrology and mat biomass, which have thus far returned mixed results (e.g., Kohler et al., 2015c; Gooseff et al., 2017).

Diatoms are single-celled, siliceous, eukaryotic algae that are useful model organisms for studying stream processes due to their ubiquity, species-specific habitat preferences, and relative ease in morphological identification (e.g., Brown and Swan, 2010; Soininen and Teittinen, 2019; de Oliveira et al., 2020). Their localized habitat preferences also make diatoms effective tracers for identifying the sources of transported materials in riverine catchments (Pfister et al., 2017). MDV microbial mats and sediments host a well-described flora of about 50 diatom species, many of which are endemic to Antarctica (Esposito et al., 2008; Kohler et al., 2015b; Sakaeva et al., 2016; Kociolek et al., 2017). Diatom communities here are structured by physical processes, including intra-and inter-annual hydrology (Esposito et al., 2006; Stanish et al., 2011, 2012; Kohler et al., 2015a), as well as historical processes such as dispersal (Sakaeva et al., 2016; Sokol et al., 2020; Schulte et al., 2022). Furthermore, MDV diatom species seem to have preferences for some mat types over others (Schulte et al., 2022) which makes it possible to use diatoms as tracers for the mobilization of different mat types. Previously, diatoms present in hyporheic sediments were found to be most similar to black mat diatom communities (Heindel et al., 2021), leading to the hypothesis that mat-derived diatoms in the POM are retained in stream sediments following hyporheic exchange. This scenario would not only potentially account for the elevated content of biogenic silica (2–8%, Heindel et al., 2021) and nitrogen (~40 g N m^−2^, Singley et al., 2023) found in the hyporheic sediment, but would also have implications for MDV diatom dispersal.

Here, we test whether different stream habitat types constitute different proportions of the transported POM diatom assemblages at different points along daily flow cycles. To do this, we collected temporally-integrated POM samples during daily flow cycles over three summers that spanned a range of flow conditions in Von Guerard Stream, Taylor Valley. To link bulk diatom transport with hydrology, we identified and quantified diatom cells in POM and compared the results with stream discharges. We then exploited differences in the diatom communities between habitat types (black mats, orange mats, and bare sediments) to estimate their proportional contribution to the POM diatom assemblage at different points on the hydrograph by using a Bayesian SourceTracker mixing model (Knights et al., 2011). We hypothesized that different MDV habitat types will differ in their potential for mobilization given their in-stream position, dictating both their proportional representation in the POM diatom assemblage, and potentially also the diatom assemblage retained in hyporheic sediments.

## 2 Methods

### 2.1 Site description

**Figure 1:**
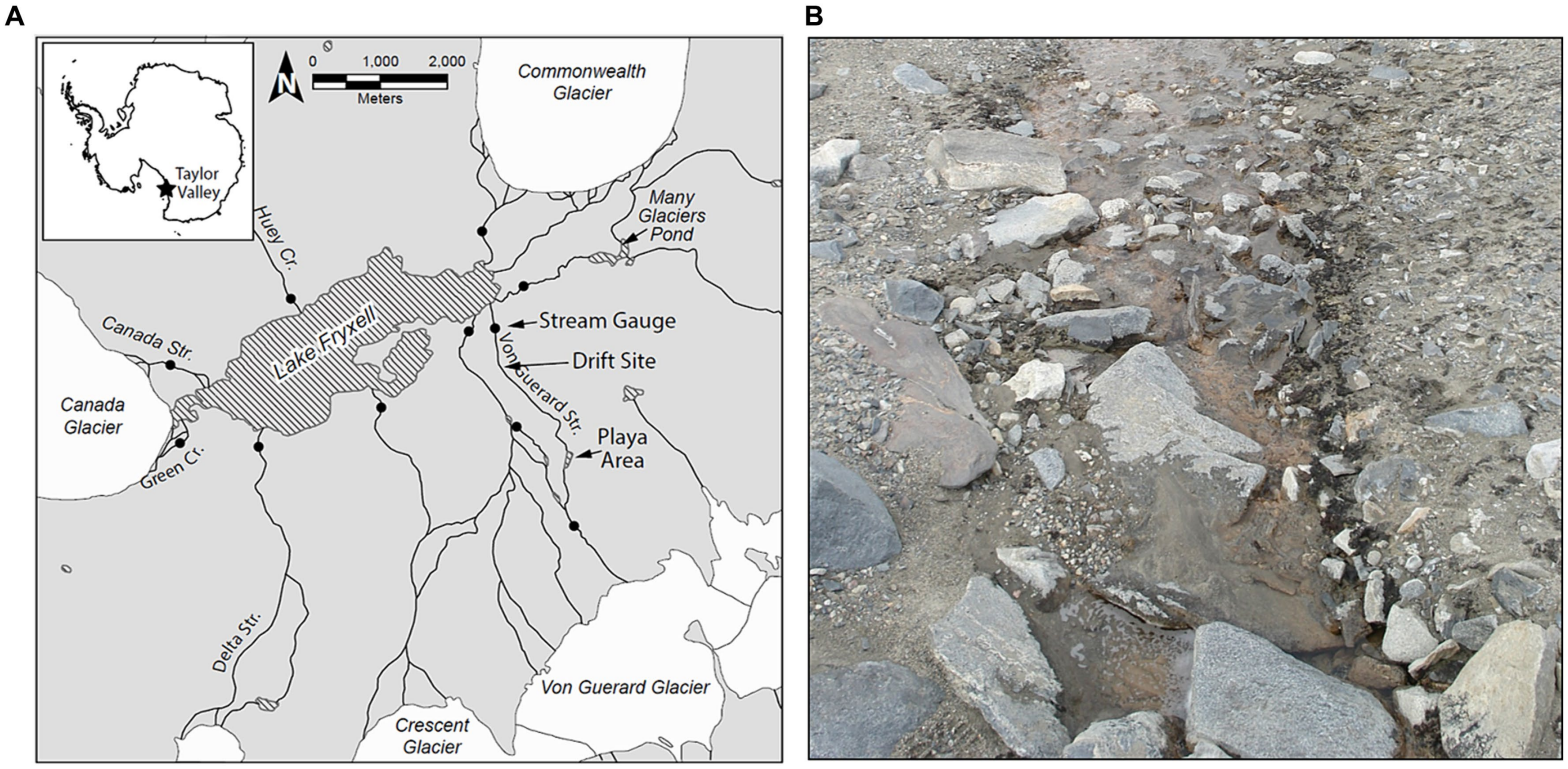
**(A)** Overview map of the Lake Fryxell Basin within Taylor Valley, McMurdo Dry Valleys, Antarctica. Closed circles indicate locations of stream gauges operated by the MCMLTER. Arrows indicate the location of the Drift experiment site, as well as the locations of the ‘playa’ area and the Von Guerard Stream Gauge. **(B)** Typical presentation of microbial mats in a Dry Valley stream (Bowles Creek, near Green Creek in the Fryxell Basin). Black ‘mats’ of nitrogen-fixing *Nostoc* occupy stream margins, while orange microbial mats carpet the stream benthos.

The MDV landscape features numerous alpine and terminal glaciers that feed meltwater streams which discharge (for only ~4–12 weeks during the austral summer) into the perennially ice-covered lakes on the valley floors (Fountain et al., 1999). Of these streams, we conducted experiments within Von Guerard Stream in Taylor Valley (Figure 1A). Von Guerard is the second-longest stream draining into Lake Fryxell, and has been the site of numerous research projects by the McMurdo Long-term Ecological Research Project (MCMLTER, mcmlter.org; McKnight et al., 1998, 2004; Cullis et al., 2014; Kohler et al., 2018; Heindel et al., 2021; Singley et al., 2021). Discharge here has been monitored for almost three decades, and because of its length, it is typically one of the last streams in the basin to commence flow in summer. When flowing, Von Guerard exhibits a pronounced discharge peak 6–8 h after sunlight hits the source glacier, which increases discharge up to 10-fold (Conovitz et al., 1998).

The POM transport studies (hereafter referred to as “Drift” experiments) were conducted in a lower reach of this stream (−77.613889, 163.259444; Figure 1A), which has a low gradient and stable stone pavement substrata (McKnight et al., 1999; Cullis et al., 2014). Orange mats are abundant in sections of the main channel and patches of black mats occur along the margins, representing the majority of the standing biomass (e.g., Figure 1B). An expansive area of saturated bare sediment, referred to as “the playa,” is located ~3 km upstream of the experimental reach (McKnight et al., 2007; Figure 1A), and is representative of the many backwaters and mat-free “sediment zone” areas downstream. Microbial mat coverage in the playa is sparse, and at most consists of fine orange-pigmented biofilms as opposed to the thick cohesive mats found further downstream.

### 2.2 Experimental design and sampling

To investigate potential differences in POM sources over daily flow cycles, we sampled POM over four time periods taking place within three summers as previously outlined in detail by Cullis et al. (2014): “Drift 1” (22–23 January 2008), “Drift 2” (17–19 January 2009), “Drift 3” (22–23 January 2009), and “Drift 4” (20–22 January 2011). For all four Drift experiments, POM was collected using a plankton net (40 μm mesh) with a 10 μm mesh collection bucket (Wisconsin bucket, Wildco, Yulee, FL, United States) that was anchored to the streambed and deployed in the stream for an average of 4.6 h (range = 2.2–10.1 h). Following deployment, trapped POM was rinsed into the collection bucket, and the subsequent slurry was divided into aliquots which were analyzed for chlorophyll *a* (Chl-*a*), ash-free dry mass (AFDM), and diatom cell counts and community characterization. At each time-point, water temperature and pH were measured, and bulk water samples were collected and analyzed for nutrients (i.e., NH_4_^+^, NO_3_^−^, and PO_4_^3−^) as outlined in Welch et al. (2010). Bulk water samples were also collected opportunistically at the downstream Von Guerard gauging station within the same summers (although outside of the Drift experiment periods), and analyzed for Si according to Welch et al. (2010). Continuous measurements (every 15 min) of stream discharge were recorded at the Von Guerard Stream Gauge, and air temperature, wind speed, and photosynthetically active radiation (PAR) were obtained from a nearby meteorological station. All discharge, meteorological, and water quality data are available online at mcmlter.org.

### 2.3 Diatom quantification and characterization

Samples for Chl-*a* were filtered using 25 mm pre-combusted GF/C filters and stored frozen until analysis. Chl-*a* samples were extracted in buffered acetone and analyzed using a Turner Designs 10-AU fluorometer (Turner Designs, Sunnyvale, CA) according to Welschmeyer (1994). The concentration and flux of POM was calculated using equations outlined in Cullis et al. (2014), who used the same set of samples to model POM flux dynamics. Samples for diatom quantification were settled and preserved in a 5% formalin solution. Using this preserved material, the percent live (presence of intact protoplasm in cells) and dead diatoms (empty cells) were determined in samples that had sufficient material for quantitation: five Drift 1 samples, one Drift 2 sample, and six Drift 3 samples; percent live measurements were not made for the Drift 4 samples due to a lack of preserved material.

For Drift 1 (*n* = 6), Drift 3 (*n* = 6), and Drift 4 (*n* = 10), an aliquot of each POM sample was taken to quantify both relative and total diatom species abundances. Due to low biomass, only one Drift 2 diatom sample could be enumerated, and this experiment is therefore not discussed further in detail. At each processing step, the percent of the total sample was recorded in order to keep all results quantitative. Aliquots were digested using heat and H_2_O_2_ and rinsed several times with distilled water to return to a neutral pH. A known volume of the digested material was then allowed to dry evenly onto coverslips, which were then permanently mounted onto glass microscope slides with the mounting medium Zrax (W. P. Dailey, Philadelphia, United States). Diatom enumerations were performed in transects, taking note of both the number of cells and the fields of view counted, using an Olympus Vanox light microscope (Japan) at 1250× magnification. The Antarctic Diatoms Website^1^ and citations therein served as the basis for species determinations. Diatom biovolumes were further calculated for each taxon individually using the methodology outlined in Stanish et al. (2011). Quantitative diatom counts were subsequently scaled to the volume of sampled streamwater (diatom cells L^−1^ and diatom biovolume L^−1^) using equations found in Cullis et al. (2014).

To estimate potential sources of POM from different in-stream habitats, diatom community composition was determined from black mats, orange mats, and bare sediment “playa” samples from Von Guerard Stream. Specifically, we used relative abundance counts from orange and black mat samples collected during the 1993–1994, 2002–2003, 2006–2007, 2007–2008, and 2009–2010 summer seasons, and from sediments collected in 2012–2013 and 2018–2019 from the outlet of the playa, to compare with POM samples (Supplementary Table S1). The playa samples were included to represent and assess the contribution of diatoms that do not necessarily inhabit microbial mats, but may occupy sediments in low-flow, depositional areas. In addition, particular effort was made to accurately count cells of small and lightly silicified diatoms such as *Fistulifera pelliculosa*. This taxon is subject to under-reporting due to its propensity to being overlooked on a densely-covered, sediment-laden microscope slide. Thus, samples from Von Guerard Stream used for comparison with Drift experiment samples were carefully re-analyzed to accurately determine the presence of *F. pelliculosa*. Count data are available at the Antarctic Freshwater Diatoms website (see Footnote 1).

### 2.4 Statistical analyses

Non-metric multidimensional scaling (NMDS) analysis was used to visualize the relationships between POM-associated diatoms and their potential sources using the *vegan* package (Oksanen et al., 2018) in R (R Core Team, 2022). A Bray-Curtis distance matrix was generated from diatom counts with rare taxa excluded (taxa whose maximum relative abundances were <2% across all samples), and a 3-dimensional model produced a goodness-of-fit value of 0.095 using Kruskal’s stress formula. A Shepard plot of calculated vs. raw dissimilarities showed strong non-metric (*r*^2^ = 0.99) and linear (*r*^2^ = 0.95) fits. A PERMANOVA analysis of diatom community differences across potential POM sources and Drift experiments was performed using the *adonis2* function in *vegan*. We furthermore conducted an indicator species analysis using the *indicspecies* R package (De Caceres and Legendre, 2009) to statistically identify species characteristic of a particular habitat type by using the un-filtered and un-transformed relative abundance data (with association function = IndVal.g, duleg = TRUE).

The relative contribution of different habitat/mat types to the POM was assessed using the Bayesian probability modeling software SourceTracker version 0.9.1 (Knights et al., 2011), in R (R Core Team, 2022). This software was designed for DNA sequencing studies to identify sources of contamination in large datasets based on user-defined source communities, and has previously been applied to identify the source of diatoms within MDV streams (Heindel et al., 2021). The model implements Gibbs sampling to evaluate the uncertainty associated with the model output for each sample. A set of priors are used to account for unknown probability distributions in the model and are detailed in Knights et al. (2011). Briefly: alpha1 represents the prior for counts of each species in the training environments; alpha2 is the prior for counts of each species in the Unknown environment; and beta is the prior for counts of test sequences in each environment. To determine the values to use for the priors, models were generated using the default values and three alternative sets of values for alpha2 and beta (the model was relatively insensitive to changes in alpha1). The model was trained using diatom relative abundance counts representing bare sediments (i.e., from sediments collected from the outlet of the playa) and orange and black mats (from samples collected from a nearby long-term microbial mat transect site on Von Guerard Stream; Supplementary Table S1). Representative samples from each habitat were selected using ordination analysis, and were chosen based on their proximity to the population centroid in ordination space. A similar number of samples from each habitat was used to reduce bias in the training data set. The best model was determined by comparing the predicted vs. actual source proportions for source samples. Using these criteria, the optimized parameters were: alpha1 = 0.001, alpha2 = 0.1, and beta = 0.1.

Results from the SourceTracker models thus represent the proportion of individual sources composing the POM diatom assemblages. However, these results alone can be potentially misleading out of context, given that (1) diatom cells differ in their streamwater concentration over time, (2) the size distribution of the diatom cells (and by extension diatom biovolumes) varies with assemblage composition, and (3) stream discharge varies within and across summers. Thus, to further explore trends within these data, proportional results from the SourceTracker analysis were further weighted based on (1) diatom cell concentrations in streamwater (to estimate differences in diatom cells L^−1^ by source), (2) average diatom biovolume for a given source community (to estimate differences in μm^3^ diatoms L^−1^ by source), and (3) stream discharge at the Von Guerard Gauge (to estimate differences in μm^3^ diatoms s^−1^ by source).

## 3 Results

### 3.1 Experimental conditions

**Figure 2:**
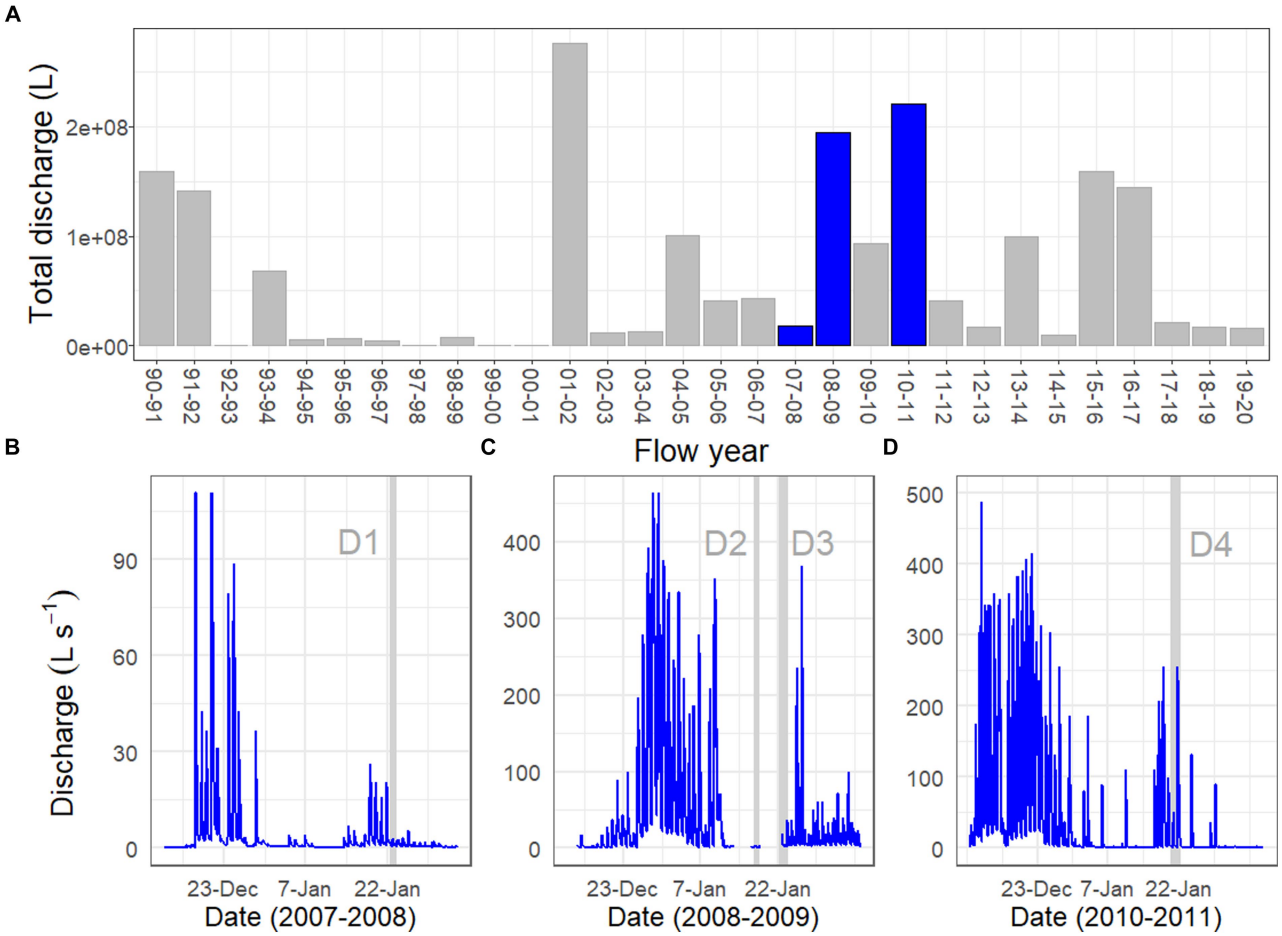
Discharge records for Von Guerard Stream at the gauge: **(A)** total streamflow measured for each summer flow year, starting from 1990 to 1991 and ending with 2019–2020. Blue bars indicate flow years investigated in this work (2007–2008, 2008–2009, and 2010–2011). Bottom panels are continuous discharge measurements during the **(B)** 2007–2008, **(C)** 2008–2009, and **(D)** 2010–2011 flow years. Vertical gray bars within these panels indicate the time intervals for the Drift experiments (the ‘D1–D4’ labels refer to ‘Drift 1–Drift 4’). Note differences in *y*-axis scales across the three flow years.

Drift samples were collected during one summer of relatively low flow (2007–2008; Drift 1) and two summers of sustained high flows (2008–2009, 2010–2011, Drift 2–4). These flow regimes roughly span the range of flows recorded since the beginning of the flow record in 1990–1991 (Figure 2A), and included several daily flow peaks which exceeded 100 L s^−1^, a threshold which has been considered to represent a potential scouring event for mat biomass (Esposito et al., 2006; Cullis et al., 2014). The total annual flow volume in Von Guerard Stream during the 2007–2008 season (Drift 1) was ~1/3 the historic mean measured from 1990 to 2020. Meanwhile, total streamflow in the 2008–2009 flow season was ~3 times higher than the 1990–2020 average and was the third highest on record (Figure 2A). Finally, total streamflow in the 2010–2011 season was slightly higher than the 2008–2009 season and was ~3.5 times higher than the historic average, making its total discharge (currently) second only to the ‘flood’ year of 2001–2002 (Figure 2A).

**Figure 3:**
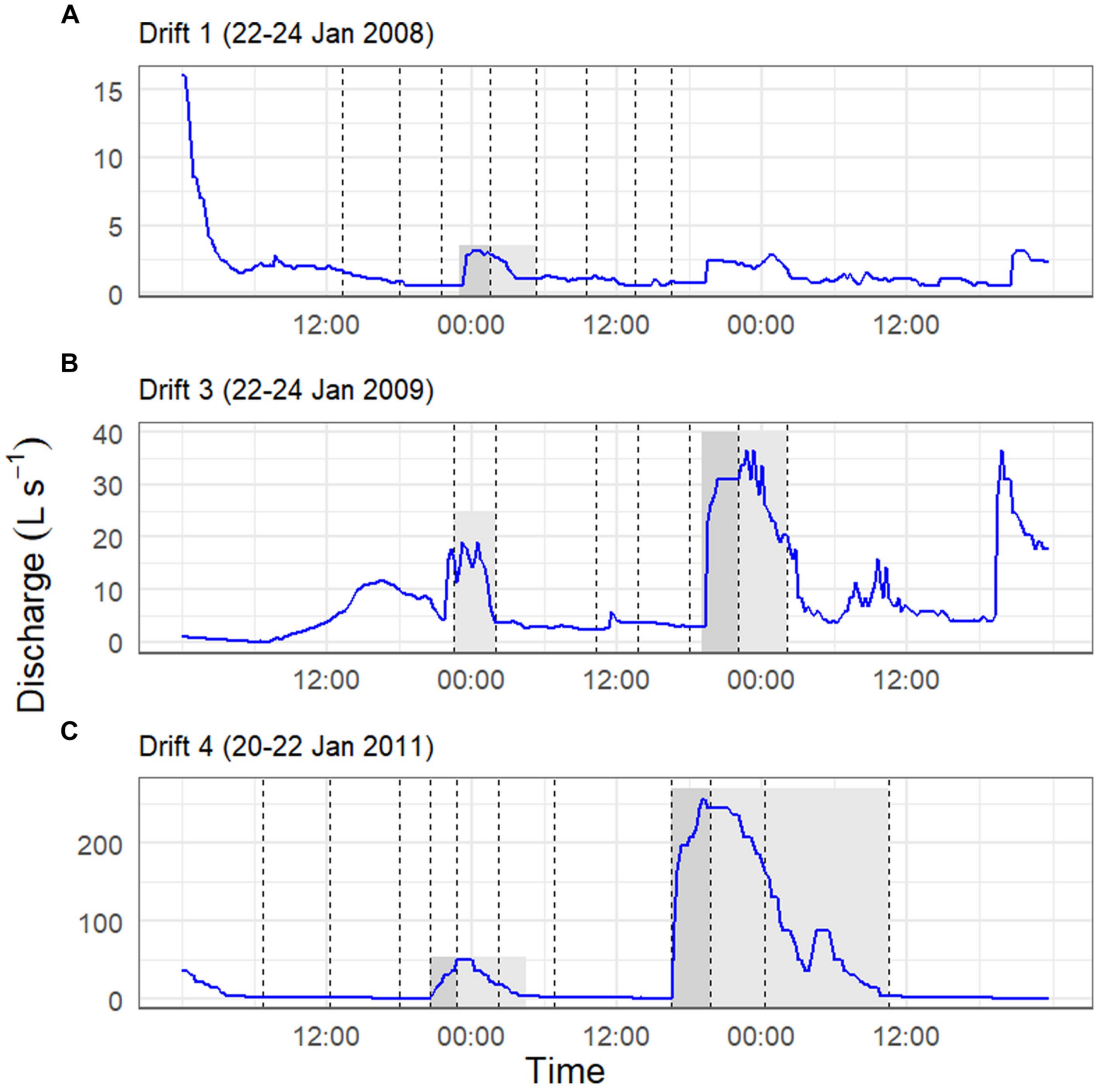
Stream discharge (*y*-axis) over a two-day time period (*x*-axis) for the Drift 1 **(A)**, Drift 3 **(B)**, and Drift 4 **(C)** experiments. Drift 2 is not depicted given that it lacked a daily flow peak. Gray shaded areas during sampling periods highlight time intervals associated with the daily flow peak, with dark gray indicating the rising limb of the hydrograph, and the light gray indicating the falling limb. Non-shaded sections of the hydrograph during the sampling periods were considered to represent ‘baseflow’ conditions. Dashed vertical lines correspond to net collection periods. Notice the difference in scales between the three *y*-axes.

The physical conditions present prior to, and at the time of, each Drift experiment varied considerably (Table 1; Supplementary Figure S1). For Drift 1, sampling occurred at the end of a week of modest flows, with daily flow peaks reaching less than 30 L s^−1^ (Figure 2B). Flows reaching 100 L s^−1^ had occurred over a few days in mid-December, but were followed by lower flow conditions (Figure 2B). While the daily flow cycle over which Drift 1 was conducted most closely represented what can be considered ‘typical’ (i.e., occurring approximately 8 h after the source glacier received direct sunlight), it represents the smallest flood pulse of our study (excluding Drift 2), with a flow peak of ~3 L s^−1^ (Figure 3A).

**Table 1:** Physical and chemical conditions during each Drift experiment.

	Air Temp (°C)			PAR (μmol s^−1^ m^−2^)			Water Temp (°C)			Q (L s^−1^)			Nutrients (μg L^−1^)		
	Max	Min	Mean	Max	Min	Mean	Max	Min	Mean	Max	Min	Mean	NH_4_^+^	NO_3_^−^	PO_4_^3−^
Drift 1 (22–24 Jan 2008)	0.1	−3.7	−1.7	1,393	91	627	10.1	0.1	3.6	3.1	0.6	1.2	2.7 (±0.8)	4.1 (±0.3)	7.4 (±0.2)
Drift 2 (18–20 Jan 2009)	−1.0	−6.7	−3.2	1,133	131	624	5.7	0.3	3.3	1.8	~0	1.5	1.7 (±0.3)	42.6 (±3.7)	15.2 (±0.6)
Drift 3 (22–24 Jan 2009)	1.6	−5.2	−1.4	1,111	88	676	9.3	0.1	4.4	36.5	2.2	9.4	1.9 (±0.3)	21.3 (±4.2)	12.4 (±0.3)
Drift 4 (21–22 Jan 2011)	0.6	−6.1	−0.7	1,533	82	581	10.2	0.3	4.9	256.2	1.3	52.8	3.8 (±3.7)	42.5 (±86)	15.4 (±4.4)

Nutrient values represent the mean of measurements made throughout the sampling period, with standard deviations in parentheses. For Drift 4, no water samples were taken during the experiment, and therefore reported nutrient concentrations are averages from the Von Guerard Stream Gauge for the entire summer (*n* = 5). PAR, photosynthetically active radiation, and Q = discharge extrapolated from continuous measurements at the Von Guerard Stream Gauge.

By contrast, the diel flow cycles sampled in 2008–2009 (Drift 2 and Drift 3) occurred approximately 1 week after a 16-day period of sustained high flows, with discharges commonly exceeding 100 L s^−1^ during this time, and two daily flow peaks reaching discharges >450 L s^−1^ (Figure 2C). However, cloudy conditions set in, and when Drift 2 was conducted, air temperatures were low, and no distinct flow peak was observed. Drift 3 was conducted several days later with the onset of an extended period of warm temperatures associated with strong foehn-like winds (Table 1; Supplementary Figure S1). Consequently, both of the daily flow peaks encapsulated during Drift 3 (peaking at 19 and 36 L s^−1^, respectively) were larger than for Drift 1 (and much larger than for Drift 2), and a third flow peak (albeit much smaller than the main two) even occurred midday on 23 January (Figure 3B).

Drift 4, which took place during the 2010–2011 season, also captured two consecutive daily flow peaks, and occurred after a four-day period when flows exceeded approximately 100 L s^−1^ (Figure 2D). During the Drift 4 experiment, winds were relatively calm, and this period had the highest peak PAR values and mean water and air temperatures among the four experiments (Table 1; Supplementary Figure S1). Accordingly, the Drift 4 daily flow peaks, particularly on the second day, were the largest of the four experiments, with the flow peak during Drift 4 reaching 51 L s^−1^ on the first day and 256 L s^−1^ on the second (Figure 3C). After the flow peak had receded, the orange mats on the streambed appeared to have been shredded due to scour (McKnight, personal observation).

In contrast to the hydrologic conditions, the concentrations of NH_4_^+^ and NO3-averaged over the sampling windows were comparable across experiments. The NH_4_^+^ concentrations generally were below 5 μg L^−1^, NO3-concentrations were between 4 and 43 μg L^−1^, and PO43-concentrations between 7 and 15 μg L^−1^ (Table 1). Bulk water samples collected from downstream Von Guerard and analyzed for Si showed consistency in their concentrations within and across summers. Mean (and standard deviation) values for the relevant seasons include 2.21 mg Si L^−1^ (SD ± 0.58) for 2007–2008 (*n* = 10), 2.80 mg Si L^−1^ (SD ± 0.29) for 2008–2009 (*n* = 6), and 2.25 mg Si L^−1^ (SD ± 0.32) for 2010–2011 (*n* = 6).

### 3.2 POM-associated diatom dynamics

**Figure 4:**
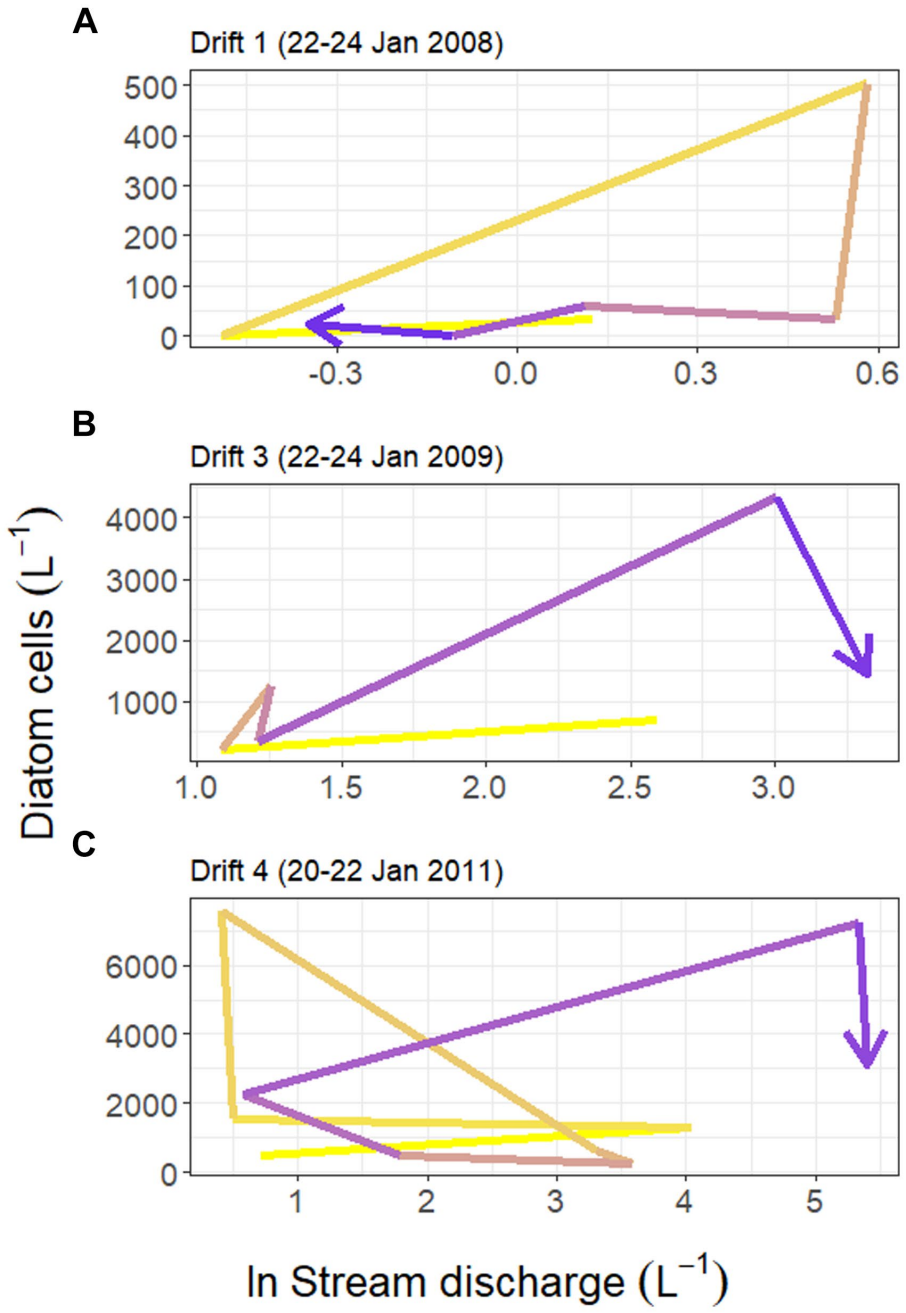
Hysteresis in the relationship between Von Guerard Stream discharge (measured at the downstream gauge, natural log transformed) and concentrations of diatom cells in the Drift 1, Drift 3, and Drift 4 experiments during each time period (panels **A–C**, respectively). Yellow shades correspond to earlier time periods in the hydrograph, and blue colors correspond to later periods. Notice the difference in scales between the three *y*-axes.

Substantial differences were present in the concentration of diatom cells mobilized throughout each daily flow cycle and across Drift experiments. Diatom cell concentrations were lowest during Drift 1, with an average of 94.1 (SD ± 182.5) cells L^−1^, and highest during Drift 4 with 2,491.7 (SD ± 2,734.9) cells L^−1^ (Table 2). There was a non-linear increase in diatom concentrations in relation to discharge on an inter-seasonal basis. For example, the average discharge of Drift 3 was ~10× greater than in Drift 1, and produced ~15× more diatom cells L^−1^. Meanwhile, the average discharge of Drift 4 was ~50× greater than Drift 1, but only 26× more diatoms L^−1^. Similarly, the average discharge from Drift 4 was ~5× greater than Drift 3, but only produced on average 2× more diatoms L^−1^. Intra-seasonally, a clockwise hysteresis pattern was observed in the total diatom cell densities (Figure 4), and this pattern was reflected by nearly every diatom taxon for which abundances could be accurately measured (Supplementary Figure S2).

**Table 2:** Mean biomass values measured as ash-free dry mass (AFDM), diatom cells, and chlorophyll *a* (Chl-*a*) per liter of streamwater for each drift experiment.

Experiment	*n*	AFDM (mg L^−1^)	Diatom cells (L^−1^)	Chl-*a* (ug L^−1^)	Chl-*a*: AFDM	Cells: AFDM	Cells: Chl-*a*
Drift 1	7	5.11 × 10^−3^	94.1	5.59 × 10^−3^	1.01	18,424	16,824
Drift 3	6	3.46 × 10^−3^	1,387.8	2.53 × 10^−3^	0.73	401,453	547,935
Drift 4	10	3.45 × 10^−4^	2,491.7	3.39 × 10^−5^	0.10	7,214,375	73,411,600

Also given are the ratios of Chl-*a*: AFDM, diatom cells: AFDM, and diatom cells: Chl-*a*. For Drift 1, diatom cells were quantified from *n* = 6 rather than *n* = 7.

To investigate relationships between biomass and hydrology further, diatom viability counts were performed on Drift 1 and Drift 3 samples, which showed that the condition of diatom cells varied during each experiment. During Drift 1, most of the diatom cells had visible chloroplasts (mean = 75%, range = 71–90% viability), while dead cells were more common in Drift 3 (average = 57%, range = 51–72% viability). Average viable cell concentrations were thus 70 and 795 cells L^−1^ for Drift 1 and Drift 3, respectively, and the ratio of live diatom cells to both AFDM and Chl-*a* was much higher during Drift 3 than during Drift 1. The same was true when the ratio of total diatom cells (both alive and dead) to both AFDM and Chl-*a* were calculated; Drift 3 was a full order of magnitude greater than Drift 1, and Drift 4 two orders of magnitude greater than Drift 3 (Table 2). Thus, changes in the magnitude of discharge inter-seasonally influenced the stream’s capacity to remove diatom cells from the substrata, above and beyond that of stream Chl-*a* and AFDM (most of which is likely derived from cyanobacteria).

The relative abundance of individual diatom taxa varied across habitat types (Supplementary Figure S3), and an indicator species analysis was conducted to identify the most representative taxa for each habitat type (Supplementary Table S2). Specifically, for black mats, 11 taxa were identified as indicators, with the highest-ranking three being *Hantzschia amphioxys* f. *muelleri*, *Hantzschia* spp., and *Navicula seibigiana*. For orange mats, the three best indicator taxa (out of four total) were *Stauroneis latistauros*, *Craticula molestiformis*, and *Luticola laeta*. For the bare sediment “playa” samples, the three best indicator taxa (also of four total) were *Amphora oligotrophenta, Fistulifera pelliculosa,* and *Mayamaea atomus*. The latter two of these, namely *F. pelliculosa* and *M. atomus*, are very lightly silicified and among the smallest known diatom taxa in the MDV, reaching only ~10 μm in maximum length (Supplementary Figure S4). As a result, the average biovolume per diatom cell differed considerably between the different habitat types, being 437 μm^3^ for orange mats, 467 μm^3^ for black mats, and 274 μm^3^ for the playa sediments. Meanwhile, there were no significant indicator taxa for POM samples. Yet, it must be noted that *F. pelliculosa* was the most abundant member of POM assemblages, garnering an average of 31% of the relative abundance across all POM samples (Supplementary Figure S3), followed by species of *Hantzschia* (*H. abundans* and *H. amphioxys*) and *Luticola* (*L. austroatlantica*, *L. muticopsis*, and *L. laeta*) each with an average of ~7–8% of the relative abundance.

**Figure 5:**
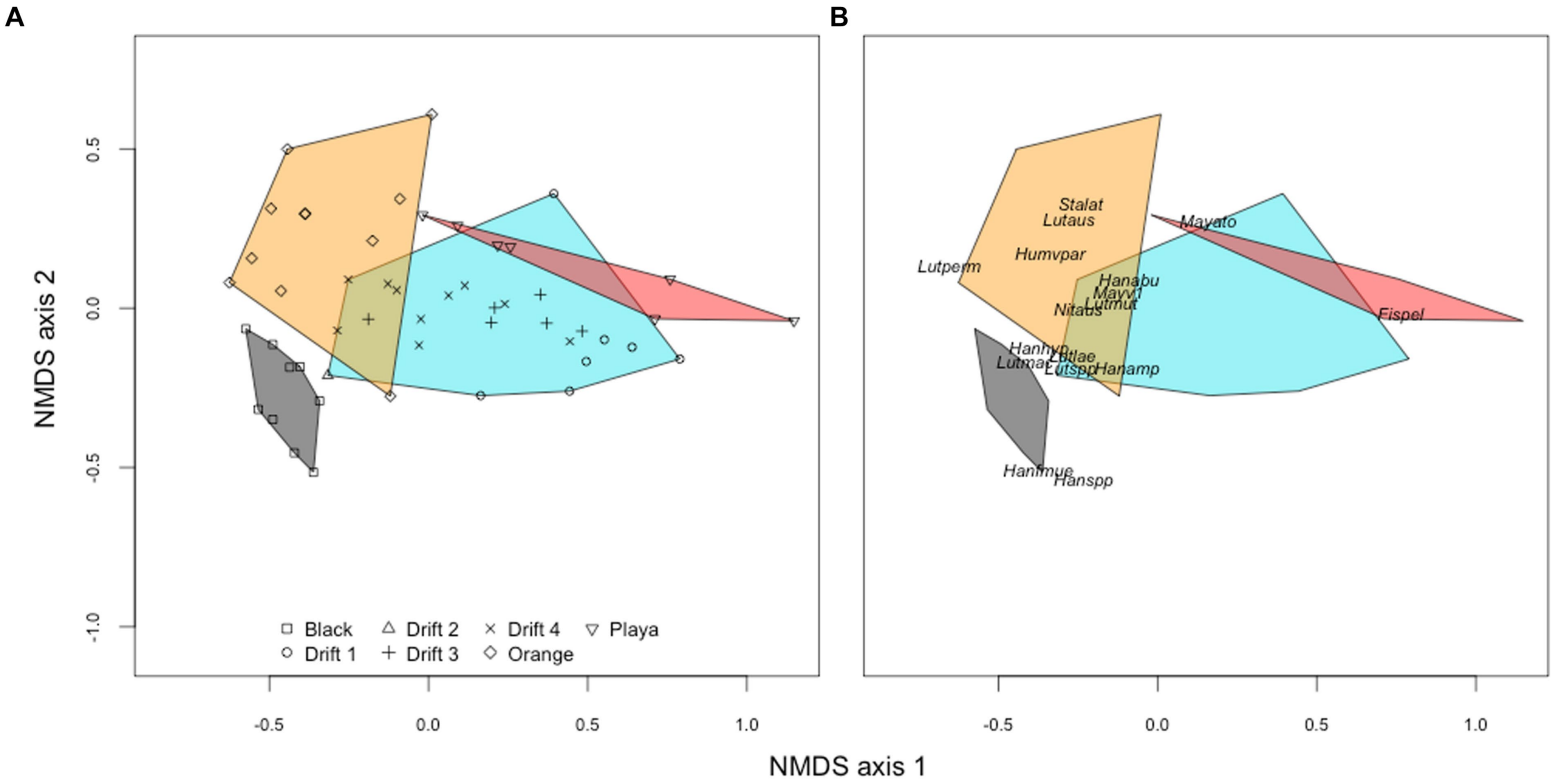
NMDS ordination (Bray–Curtis distances) of diatom communities from orange mats (orange), black mat (black), and bare ‘playa’ sediments (red) plotted alongside POM assemblages corresponding to the four Drift experiments (blue). The left panel **(A)** shows the orientation of individual samples to each other, while the right panel **(B)** shows the placement of common taxa (minimum abundance of 5% in at least one sample). Abbreviations for panel **(B)** are as follows: Ach_tay, *Achnanthes taylorensis*; Fis_pel, *Fistulifera pelliculosa*; Han_abu, *Hantzschia abundans*; Han_amp, *H. amphioxys*; Han_hyp, *H. hyperaustralis*; Han_f_mue, *H. amphioxys* f. *muelleri*; Han_spp, *Hantzschia* sp.; Hum_v_par, *Humidophila arcuata* var. *parallela*; Lut_aus, *Luticola austroatlantica*; Lut_lae, *L. laeta*; Lut_mac, *L. macknightiae*; Lut_mut, *L. muticopsis*; Lut_per, *L. permuticopsis*; May_ato, *Mayamaea atomus*; May_v_1, *M. atomus* var. 1; May_v_per, *M. atomus* var. *permitis*; Nav_sei, *Navicula seibigiana*; Sta_lat, *Stauroneis latistauros*.

To better visualize the relationships among diatom assemblages across habitat types, we created an NMDS ordination. On the first ordination axis (NMDS1), *F. pelliculosa* strongly drove differences between the POM and playa samples from the orange and black mats (Figure 5A). The second ordination axis (NMDS2) separated orange and black mats, primarily due to differences in the relative abundances of genera *Hantzschia* and *Luticola* (Figure 5B). There were significant global differences in diatom assemblages across POM, mat, and playa samples (PERMANOVA, Pseudo-*F* = 10.63, *R*^2^ = 0.404, *p* < 0.001), and across the three potential POM sources (i.e., black mats, orange mats, playa sediments) when POM samples were excluded from the analysis (Pseudo-*F* = 9.92, *R*^2^ = 0.467, *p* < 0.001).

The SourceTracker program provided a means to quantitatively resolve the source of mobilized diatom taxa from the different habitat types over daily flow cycles. Overall, bare ‘playa’ sediments represented a major proportion of diatoms to the POM at all time points and summers (Supplementary Figure S5; Table 3), averaging 56% across all experiments (58% when excluding Drift 2). However, the proportion of playa contributions decreased with increasing total seasonal discharge; the playa sourced an average of 78% of the POM assemblages in Drift 1, 62% in Drift 3, and 41% in Drift 4. Meanwhile, orange and black mats individually contributed lower proportions to the POM diatom assemblages overall, but were similar in proportion to each other. Specifically, orange mats contributed on average 23% to the POM across experiments, while black mats composed on average 21%. However, in contrast to the playa samples, contributions from orange and black mats both increased overall from the low flow year (Drift 1) to the highest flow year (Drift 4, Table 3). Interestingly, there was an overall concurrence with the ordination results in that POM samples from Drift 4 (i.e., the experiment with the highest flow) were the most similar to the black and orange mats of all the POM samples (disregarding Drift 2). Meanwhile, Drift 1 differed the most from the mat samples, while Drift 3 was intermediate (Figure 5A).

**Table 3:** Average modeled percent contribution, and biovolume-corrected percent contribution, of channel-occupying orange mats, marginal black mats, and bare sediment ‘playa’ habitats (as well as unknown sources) to the transported POM diatom assemblages from each Drift experiment.

Experiment	*n*	Orange	Black	Playa	Unknown
**% contributions**					
Drift 1	7	0.04	0.17	0.78	0.00
Drift 2	1	0.44	0.56	0.01	0.00
Drift 3	6	0.23	0.14	0.62	0.01
Drift 4	10	0.35	0.24	0.41	0.00
**biovolume-corrected % contributions**					
Drift 1	7	0.06	0.26	0.69	0.00
Drift 2	1	0.42	0.57	0.01	0.00
Drift 3	6	0.30	0.19	0.50	0.01
Drift 4	10	0.41	0.30	0.30	0.00

**Figure 6:**
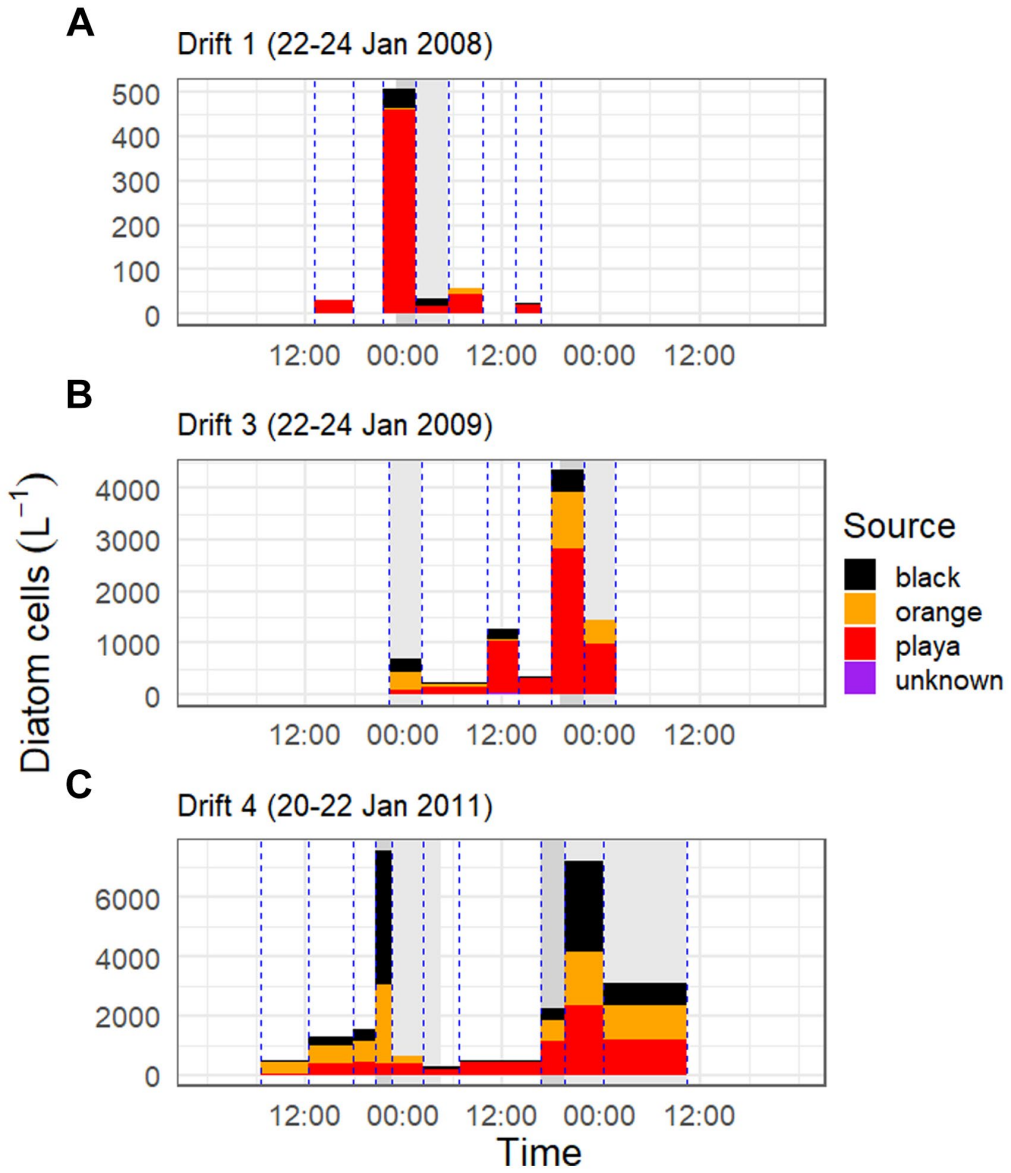
Diatom cell concentrations and Sourcetracker-modeled contributions by habitat type over the three Drift experiments [Drift 1, Drift 3, and Drift 4 are given in panels **(A–C)**, respectively]. Bar colors correspond to the proportion of the modeled source of POM (black mats = black, orange mats = orange, bare ‘playa’ sediments = red, and unknown sources = purple), and are scaled by the concentration of diatom cells in the streamwater at that timepoint. Bar width corresponds to the collection period of nets within Von Guerard Stream, and each vertical blue line corresponds with a time the collection net went in/out of the stream. Dark gray shaded areas in background indicate the rising limb of the hydrograph, and light gray bars indicate the falling limb, while other areas indicate baseflow conditions. Please note difference in y-axes between panels.

The portion of the daily flow cycle represented by a given POM sample was also found to be important in predicting its respective sources. For example, in the low flow year of Drift 1, nearly the whole POM diatom assemblage was sourced by bare sediments at all timepoints, even during the modest daily flow peak (although there was a rise in black mat contributions during this time; Figure 6A). However, in Drift 3 and 4, while the playa seemed to provide the ‘background’ source of diatoms for much of the baseflow period, large portions of the POM diatom assemblage were sourced from black and orange mats during the rising and falling limbs of the daily flow peak (Figures 6B,C). This was the most pronounced during the highest flow year (Drift 4), where orange and black mats collectively surpass the playa as the source of POM diatoms during this time (Figure 6C). The importance of the increasing contribution of orange and black mats with increasing flow is further enhanced when considering biovolume differences of the diatoms inhabiting the different habitat types (Table 3; Supplementary Figure S6), and when normalized by discharge to create the percent contributions of different sources to the total exported diatom load (Supplementary Figure S7).

When biovolume is normalized by discharge, it becomes clear that the bulk of all diatom movement in this stream takes place during these punctuated periods of high streamflow, with mats in particular being major contributors of diatoms during these times. While this is difficult to quantify accurately with our data set (i.e., given irregular POM net incubation times, difficulty in defining beginning/end of flow peaks, etc.), we nonetheless calculated the proportion of diatoms exported during daily flow peaks (gray shaded areas of hydrograph) versus baseflow conditions (areas of the hydrograph with no shading) for each Drift experiment and habitat type using discharge corrected biovolume data (Supplementary Figure S7). In doing so, we found that the overwhelming majority of diatoms are exported during the daily flow peaks. For example, during Drift 1, 78.5% of the diatom biovolume was mobilized during the daily flow peak interval (23:00–05:30), and 17.7% of the biovolume exported during this time were contributed by black mats (<1% by orange mats). Meanwhile, in Drift 3, 93.5% of all diatom biovolume was mobilized during the daily flow peak intervals (22:15–02:15 and 19:00–01:45), and 49.8% of the biovolume exported during this time was contributed by mats (12.0% black and 37.8% orange mats). Finally, for Drift 4, 99.7% of all diatom biovolume was mobilized during the two daily flow peak intervals (20:45–04:30 the first day and 16:45–10:30 the second day), and during this time, 75.2% of the biovolume originated from mats (44.0% from black mats and 31.2% from orange).

## 4 Discussion

In this study, we used diatoms as tracers to determine the contributions of different habitat types to POM diatom assemblages. By collecting POM over four ‘Drift’ experiments covering a range of hydrologic conditions in Von Guerard Stream, our findings highlight the role of hydrologic processes acting on the streambed in generating POM. Specifically, the relationship between diatom cell concentrations and stream discharge consistently produced a clockwise hysteresis pattern, showing that diatom export is source-limited in an identical manner to bulk OM (Cullis et al., 2014). Furthermore, diatoms associated with bare sediments (i.e., ‘playa’ habitats) made up a high proportion of POM assemblages at all time periods. Yet, the contribution of diatoms from black and orange mats increased during the daily flow peaks, suggesting that different habitat types may have different hydrological thresholds for their mobilization. When corrected by concentration and discharge, these results also indicate that most POM and diatom export takes place at punctuated periods within each daily flow cycle at Von Guerard Stream. These periods therefore have important implications for local biogeochemical cycling and downstream diatom dispersal.

### 4.1 In-stream sources of autochthonous POM

While much effort has been placed on distinguishing between allochthonous and autochthonous OM in stream ecosystems (e.g., Tank et al., 2010; Hotchkiss and Hall, 2015; Battin et al., 2023), much less has been done to investigate different in-stream sources of POM, despite potential differences in abundance and lability. One advantage of studying autochthonous POM sources in MDV streams is that they lack terrestrial/riparian vegetation and groundwater sources of OM, and the resident microbial communities are distinct by the habitat type they occupy (Alger et al., 1997; Kohler et al., 2015c). Here, microbial growth can be broadly grouped into orange mats located in the main channel; black *Nostoc* ‘mats’ along stream margins; and fine biofilms covering the sediments of depositional zones such as in the playa. The distinct diatom communities inhabiting these habitat types (Schulte et al., 2022) make it possible to use these diatoms to trace the relative contribution of these habitat types to overall POM assemblages transported by streamflow (Heindel et al., 2021).

In this work, we demonstrate that all three of these in-stream habitat types contribute to POM diatom assemblages. Yet, it is clear that different habitat types contribute different proportions to POM diatom assemblages depending on what part of the daily flow cycle they represent. Specifically, diatoms from bare sediment habitats were found to contribute the greatest proportion to POM diatom assemblages at lower flows – both including the baseflow periods intra-seasonally, and the lower flow years inter-seasonally. This finding is consistent with the idea that the bare sediments on which these biofilms grow are relatively easy to mobilize with limited stream energy. The same could be argued for the diatoms characteristic of these habitats as well. Specifically, *F. pelliculosa* and *M. atomus* were both indicator taxa for the bare sediment habitats, and both are very small, lightly silicified diatoms that presumably enter the POM assemblage readily.

**Figure 7:**
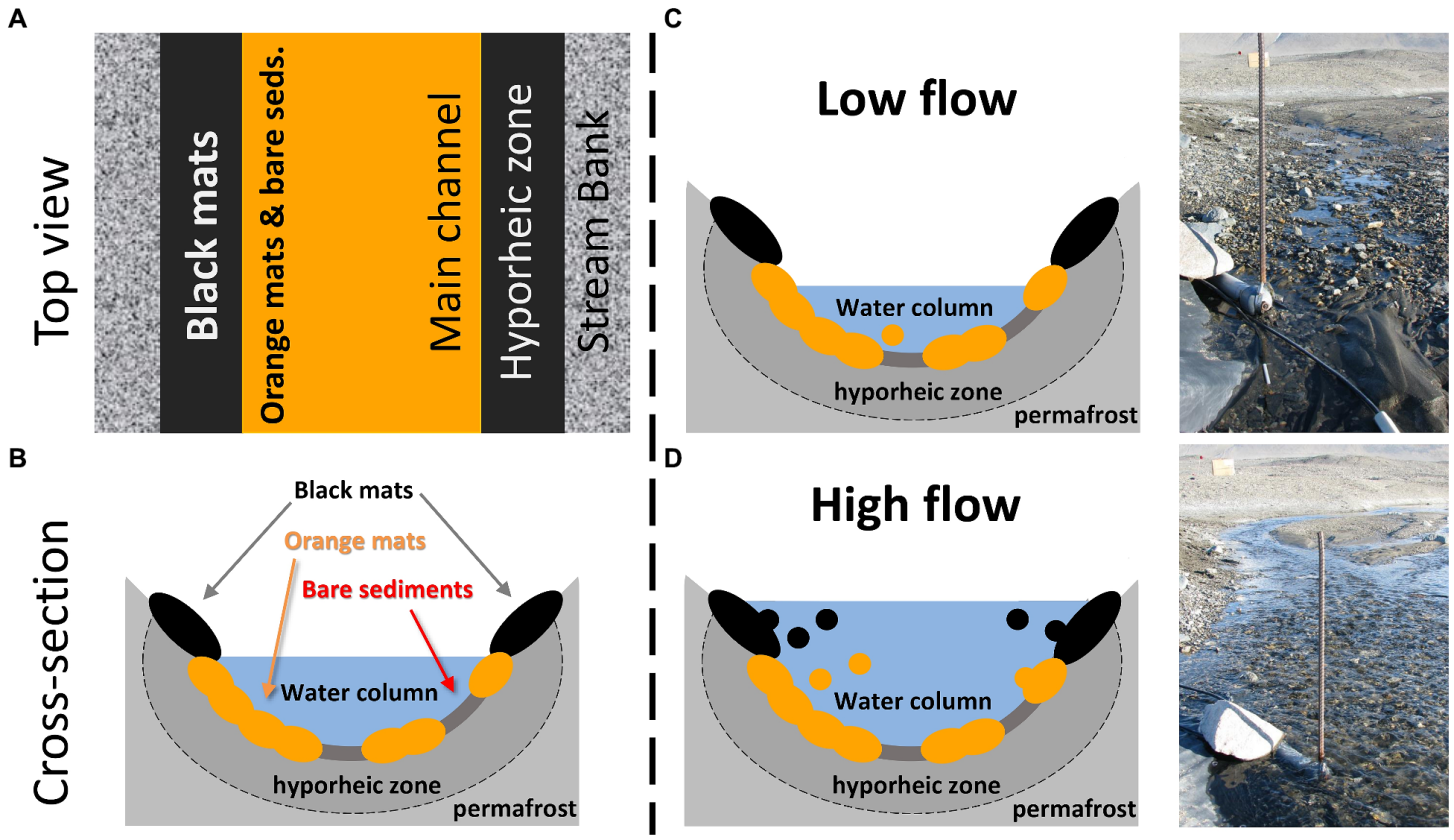
**(A)** Conceptual diagram of stream habitat locations from an overhead view of a McMurdo Dry Valley stream. **(B)** Conceptual diagram of stream habitat locations from a cross sectional view of a McMurdo Dry Valley stream. In both **(A,B)**, note the elevated and marginal position of black mats, and the lower mid-stream location of orange mats and bare sediments. **(C)** Conceptual diagram of low flow conditions with a corresponding photo of Von Guerard Stream prior to the daily flow peak. **(D)** Conceptual diagram of high flow conditions with a corresponding photo of Von Guerard Stream during the daily flow peak. Importantly, stream water inundates marginal black mats during these daily flow peaks, which are otherwise outside of the streamflow.

On the other hand, the contribution of both the orange and black mats was greater during the daily flow peaks. This is also when diatom cell concentrations were highest, indicating that, when integrating these results across an entire summer, these mats may ultimately represent the greatest source of diatoms downstream in absolute numbers. That additional energy is required to dislodge the mats and mobilize them into the water column is consistent with our understanding of these consortia as being cohesive and somewhat resistant to scour (Kohler et al., 2015c; Cuadrado and Pan, 2018). This is particularly true for orange mats that inhabit the streambed and are presumably capable of withstanding higher flows due to their position in the main channel. Black mats, on the other hand, likely only contribute substantially when stage is sufficiently high to inundate the stream margins where they reside (Figure 7). Interestingly, the characteristic diatoms for orange (*Luticola* spp. and *S. latistauros*) and black (*Hantzschia* spp. and *N. seibigiana*) mats as determined by the indicator species analysis notably have more robust silica cell walls, both in size and density. As a result, when these diatom concentrations/fluxes are normalized by diatom biovolumes, the importance of mats to the overall POM diatom assemblages further increases.

A larger percentage of empty (likely dead) diatom cells were counted during the Drift 3 experiment than in Drift 1, suggesting that there was an additional contribution of scoured, senesced material to the water column during the larger Drift 3 flood pulse compared to the smaller Drift 1 (unfortunately, we do not have data for Drift 4). Based on the model results, this senescent material likely originated from the orange mats, given comparable proportions of marginal black mats between the two flow seasons. These orange mats are perennial, and represent multiple seasons of growth (Kohler et al., 2015a). Coupled with the excellent ability of diatom cells to preserve in sediments, orange mats are therefore almost certain to contain dead diatoms that accumulate over time. Given the nearly one order of magnitude greater maximum discharge observed for Drift 3 than for Drift 1, this may have provided the extra energy required to mobilize deeper layers of the orange mats and in turn transport this senescent material. In contrast, given the high proportion of diatoms originating from bare sediments in the POM assemblages at all discharges, we can speculate that these habitats may not accumulate empty valves to the same degree as the microbial mats, and sediments may therefore export a higher ratio of live:dead diatoms than mats.

### 4.2 Implications for biogeochemical cycling

Understanding when and how different sources of autochthonous POM are mobilized can have important implications for MDV stream communities. Firstly, given the negligible grazer biomass in the MDV streams (Treonis et al., 1999), these daily flow peaks likely represent a major loss mechanism to the standing biomass of individual habitat types, and can help us to understand long-term trends in their abundance (e.g., Kohler et al., 2015c; Gooseff et al., 2017) and functional rate processes. Secondly, this downstream flux of biomass itself may have important biogeochemical implications. Notably, Drift 1 samples had greater Chl-*a*:AFDM ratios compared to the other drift dates, which may indicate higher nutritional quality during this lower flow year for consumers than in subsequent higher flow years. In fact, aliquots of the Drift 1 experiment samples were also retained and analyzed for their carbon, nitrogen, and phosphorus (C:N:P) content and C and N isotopes, and are reported in Kohler et al. (2018). Interestingly, these POM samples had higher nutrient content in comparison to the adjacent microbial mats, suggesting that POM represents a high-quality energy source, and may also serve as a substrate for microbial colonization and subsequent nutrient immobilization. From the isotope data corresponding to these same Drift 1 samples, there was also evidence that some of this material, be it from the POM matter itself or the subsequently immobilized nutrients, is derived from nitrogen-fixing black mats. Indeed, during the flow peaks of the Drift experiments, macroscopic colonies of *Nostoc* were visibly captured in the sample nets (McKnight, personal observation).

The results of the SourceTracker analyses agree that at least a portion of the transported POM is directly derived from the black mats. Given the limited nutrients released from the glacier and lack of allochthonous inputs, this is likely an important mechanism for nutrient regeneration in Von Guerard Stream and across Taylor Valley (Kohler et al., 2018, 2023). It is thought that much of this nutrient regeneration from POM takes place in the hyporheic zone, where it can be degraded and provide nutrients via upwelling for downstream communities. Evidence for this is in the work of Heindel et al. (2021), where diatoms were directly observed in the hyporheic sediments (the identity of which correspond well to that observed for the POM in this study) and were linked with greater concentrations of nitrogen (Singley et al., 2021). Given this background, the present work provides a sort of “missing link” for patching together the observations of nitrogen fixation in the surface mats (McKnight et al., 2007; Kohler et al., 2018, 2023) and the diatoms and enhanced nitrogen concentrations in the hyporheic zone (Heindel et al., 2021; Singley et al., 2021, 2023).

In addition to carbon and nitrogen, the mobilization and transport of POM-associated diatoms has the capacity to redistribute substantial quantities of silicon. In the MDV, silicon generation is probably dominated by silicate weathering in the hyporheic zone (Maurice et al., 2002), although biological processes are further needed to fully explain isotopic signatures of dissolved silicon in the water column (Hatton et al., 2020; Hirst et al., 2020). The present work may provide yet another “missing link,” with the mobilization and subsequent subduction of POM diatom assemblages potentially explaining the presence of substantial amounts of biogenic silica in the hyporheic sediments (Heindel et al., 2021). In addition, the weathering of this biogenic silica in the hyporheic zone may contribute to dissolved silicon in the streamwater, as indicated by the analysis of silicon isotopes by Hirst et al. (2020) and Hatton et al. (2020). Taken as a whole, these findings suggest that silicon transport in these streams may be influenced by autochthonous biological processes in the stream and its hyporheic zone to a much greater extent than in streams and rivers in other regions where allochthonous watershed biogeochemical processes are dominant (Jankowski et al., 2023).

### 4.3 POM as a vector for diatom dispersal

The mobilization and transport of POM also enables the dispersal of benthic organisms to downstream habitats (Peterson, 1996). The importance of dispersal for the colonization of algal communities in particular has long been recognized (Blum, 1954; Müller-Haeckel, 1971, 1973; Muller, 1974), and has recently come to the forefront in the context of the Metacommunity Framework (Leibold et al., 2004). While aerial transport is likely to be an important feature in the MDV diatom metacommunity (Šabacká et al., 2012; Diaz et al., 2018; Schulte et al., 2022), the relative importance of aerial versus aquatic dispersal to stream communities is difficult to disentangle, and is currently a matter of discussion. However, it would be highly unlikely if this unidirectional downstream flux of diatom cells in the POM was not highly influential in these streams. Firstly, this means of dispersal relocates a large number of cells directly to the target area (i.e., cells are already delivered to the ‘right’ place), and secondly, these relocated cells are also likely to be of higher viability than those aerially transported in the lower atmosphere (e.g., Schulte et al., 2022 found a maximum of up to 15% of cells to have visible protoplasm material). As POM is deposited on the streambed and resuspended in subsequent flow pulses, mats thus receive a continuous re-supply of diatoms required to support and maintain the stream mat diversity (Sokol et al., 2020). However, it is possible that the downstream flux of diatoms could potentially lead to homogenizing diatom community structure through ‘mass effects’ (when dispersal effects outweigh other assembly mechanisms like competition and environmental selection; Leibold et al., 2004), placing great ecological importance on upstream reaches. Knowing that POM is a potent vector for diatom transport is an important first step in addressing these questions, and future work should investigate these longitudinal processes in the MDV.

One of the most abundant diatoms observed in this study was *F. pelliculosa. Fistulifera pelliculosa*, is a small and lightly silicified taxon which is often abundant in eutrophic/high nutrient waters (Kociolek and Spaulding, 2003), and can be easily re-suspended in the water column (Holland et al., 1974). A clear result from this study is the reaffirmation that *F. pelliculosa* indeed has a seemingly low resistance to flood pulses and is easily mobilized. While *F. pelliculosa* was also present in the mats, it was *very* abundant in the playa sediments, which may be in part due to the elevated nutrient concentrations present within the stream sediments in comparison to the main channel as a result of hyporheic upwelling (McKnight et al., 2004; Kohler et al., 2016; Singley et al., 2021). Taken together, this creates the argument that sediments and similar depositional areas in Von Guerard Stream are not only a major source of *F. pelliculosa* to the POM, but that the POM diatoms in general may be derived from a non-trivial proportion of ‘bare’ sediments across the hydrograph.

This said, it is important to remember that even if these bare sediments made up a relatively high proportion of the POM diatom assemblages, it is almost certain that the mats play a much larger role in total exported POM biomass when these proportions are corrected by the number of cells per unit biomass of the source material (i.e., as bare sediments lack microbial mats, they likely contribute a high number of diatom cells but a low total amount of organic material). Furthermore, while the bare sediments made up a large proportion of the POM at low flows and during times of low diatom concentrations, the microbial mats had the greater contributions to POM when diatom concentrations, and overall discharges, were high. Lastly, reducing diatom cells to ‘numbers of individuals’ effectively negates cell size differences between species. This is particularly relevant when comparing *F. pelliculosa* (up to 10 μm long, characteristic of playa samples) with species of *Hantzschia* spp. (30 to >100 μm long, characteristic of black mats). Thus, while the playa was often the proportionately-dominant source of POM diatoms over time, it is important to keep the morphology of diatoms and the hydrological context in mind. In doing so, it is clear that the mats are a non-trivial component, if not dominant, source of OM and diatoms to the POM.

## 5 Conclusion

The MDV are dynamic habitats marked by environmental extremes to which organisms must adapt for survival. In this study, we show that daily flow peaks drive the downstream transport of POM. While the loss of biomass to POM during these punctuated periods may limit OM accumulation in these streams, biomass removal and subsequent export may also act to maintain downstream microbial communities through nutrient regeneration and organismal dispersal. Interestingly, the relative contribution of the different habitat types to the POM differed over the daily flow cycles, with both black and orange mats contributing the greatest proportions under periods of high flows. This highlights the importance of daily flow peaks to accessing nitrogen-fixing black mats in particular, allowing new nitrogen to enter nutrient-limited downstream reaches. Overall, these results demonstrate a temporal dimension to the connectivity of different ecosystem compartments in the MDV, and illuminates possible mechanisms into stream biogeochemistry and longitudinal diatom dispersal.

## Data availability statement

The raw data supporting the conclusions of this article will be made available by the authors, without undue reservation.

## Author contributions

LS: Conceptualization, Data curation, Formal analysis, Investigation, Methodology, Validation, Visualization, Writing – original draft, Writing – review & editing. TK: Conceptualization, Data curation, Formal analysis, Methodology, Visualization, Writing – original draft, Writing – review & editing. JD: Data curation, Formal analysis, Investigation, Writing – review & editing. DM: Conceptualization, Data curation, Funding acquisition, Investigation, Methodology, Project administration, Resources, Supervision, Writing – original draft, Writing – review & editing.

## Footnotes

^1^ About::Antarctic Freshwater Diatoms, hostwindsdns.com

## Supplementary material

The Supplementary material for this article can be found online at:

https://www.frontiersin.org/articles/10.3389/fmicb.2024.1352666/full#supplementary-material

## References

[ref1] Alger A. S. McKnight D. M. Spaulding S. A. Tate C. M. Shupe G. H. Welch K. A.. (1997). Ecological processes in a cold desert ecosystem: the abundance and species distribution of algal mats in glacial meltwater streams in Taylor Valley, Antarctica. Occas. Pap. Colo 51, available at: https://www.colorado.edu/instaar/sites/default/files/attached-files/OP51-ECOLOGICAL-PROCESSES.pdf

[ref2] Battin T. J. Lauerwald R. Bernhardt E. S. Bertuzzo E. Gener L. G. Hall R. O.. (2023). River ecosystem metabolism and carbon biogeochemistry in a changing world. Nature 613, 449–459. doi: 10.1038/s41586-022-05500-8 36653564

[ref3] Blum J. L. (1954). Evidence for a diurnal pulse in stream phytoplankton. Science 119, 732–734. doi: 10.1126/science.119.3099.732, PMID: 13168359

[ref4] Brown B. L. Swan C. M. (2010). Dendritic network structure constrains metacommunity properties in riverine ecosystems. J. Anim. Ecol. 79, 571–580. doi: 10.1111/j.1365-2656.2010.01668.x, PMID: 20180874

[ref5] Conovitz P. A. McKnight D. M. MacDonald L. H. Fountain A. G. (1998). “Fryxell Basin, Antarctica” in Ecosystem dynamics in a polar desert: the McMurdo dry valleys, Antarctica. ed. Priscu J. C. (Washington, DC: American Geophysical Union), 93.

[ref6] Cuadrado D. G. Pan J. (2018). Field observations on the evolution of reticulate patterns in microbial mats in a modern siliciclastic coastal environment. J. Sediment. Res. 88, 24–37. doi: 10.2110/jsr.2017.79

[ref7] Cullis J. D. Stanish L. F. McKnight D. M. (2014). Diel flow pulses drive particulate organic matter transport from microbial mats in a glacial meltwater stream in the McMurdo dry valleys. Water Resour. Res. 50, 86–97. doi: 10.1002/2013WR014061 PMC11112031 38784810

[ref8] De Caceres M. Legendre P. (2009). Associations between species and groups of sites: indices and statistical inference. Ecology 90, 3566–3574. doi: 10.1890/08-1823.1 20120823

[ref9] de Oliveira P. H. F. Machado K. B. Teresa F. B. Heino J. Nabout J. C. (2020). Spatial processes determine planktonic diatom metacommunity structure of headwater streams. Limnologica 84:125813. doi: 10.1016/j.limno.2020.125813

[ref10] Diaz M. A. Adams B. J. Welch K. A. Welch S. A. Opiyo S. O. Khan A. L.. (2018). Aeolian material transport and its role in landscape connectivity in the McMurdo dry valleys, Antarctica. J. Geophys. Res. Earth Surf. 123, 3323–3337. doi: 10.1029/2017JF004589

[ref11] Esposito R. M. Horn S. L. McKnight D. M. Cox M. J. Grant M. C. Spaulding S. A.. (2006). Antarctic climate cooling and response of diatoms in glacial meltwater streams. Geophys. Res. Lett. 33:L07406. doi: 10.1029/2006GL025903

[ref12] Esposito R. M. M. Spaulding S. A. McKnight D. M. Van de Vijver B. Kopalová K. Lubinski D.. (2008). Inland diatoms from the McMurdo dry valleys and James Ross island, Antarctica. Botany 86, 1378–1392. doi: 10.1139/B08-100

[ref13] Fountain A. G. Lyons W. B. Burkins M. B. Dana G. L. Doran P. T. Lewis K. J.. (1999). Physical controls on the Taylor Valley ecosystem, Antarctica. Bioscience 49, 961–971. doi: 10.1525/bisi.1999.49.12.961

[ref14] Gooseff M. N. Barrett J. E. Adams B. J. Doran P. T. Fountain A. G. Lyons W. B.. (2017). Decadal ecosystem response to an anomalous melt season in a polar desert in Antarctica. Nat. Ecol. Evol. 1, 1334–1338. doi: 10.1038/s41559-017-0253-0 29046542

[ref15] Hatton J. E. Hendry K. R. Hirst C. Opfergelt S. Henkel S. Silva-Busso A.. (2020). Silicon isotopic composition of dry and wet-based glaciers in Antarctica. Front. Earth Sci. 8:286. doi: 10.3389/feart.2020.00286

[ref16] Heindel R. C. Darling J. P. Singley J. G. Bergstrom A. J. McKnight D. M. Lukkari B. M.. (2021). Diatoms in hyporheic sediments trace organic matter retention and processing in the McMurdo dry valleys, Antarctica. J. Geophys. Res. Biogeosci. 126. doi: 10.1029/2020JG006097

[ref17] Hirst C. Opfergelt S. Gaspard F. Hendry K. R. Hatton J. E. Welch S.. (2020). Silicon isotopes reveal a non-glacial source of silicon to crescent stream, McMurdo dry valleys, Antarctica. Front. Earth Sci. 8:229. doi: 10.3389/feart.2020.00229

[ref18] Holland A. Zingmark R. Dean J. (1974). Quantitative evidence concerning the stabilization of sediments by marine benthic diatoms. Mar. Biol. 27, 191–196. doi: 10.1007/BF00391943

[ref19] Hotchkiss E. R. Hall R. O. Jr. (2015). Whole-stream 13C tracer addition reveals distinct fates of newly fixed carbon. Ecology 96, 403–416. doi: 10.1890/14-0631.1 26240862

[ref20] IPCC (2019). “IPCC special report on the ocean and cryosphere in a changing climate” in eds. Pörtner H.-O. Roberts D. C. Masson-Delmotte V. Zhai P. Tignor M. Poloczanska E. (Cambridge, UK and New York, NY, USA: Cambridge University Press), 755. Available at: https://www.ipcc.ch/srocc/cite-report/

[ref21] Jankowski K. J. Johnson K. Sethna L. Julian P. Wymore A. S. Shogren A. J.. (2023). Long-term changes in concentration and yield of riverine dissolved silicon from the poles to the tropics. Global Biogeochem. Cycles 37:e2022GB007678. doi: 10.1029/2022GB007678

[ref22] Knights D. Kuczynski J. Charlson E. S. Zaneveld J. Mozer M. C. Collman R. G.. (2011). Bayesian community-wide culture-independent microbial source tracking. Nat. Methods 8, 761–763. doi: 10.1038/nmeth.1650 21765408 PMC3791591

[ref23] Kociolek J. P. Kopalová K. Hamsher S. E. Kohler T. J. Van de Vijver B. Convey P.. (2017). Freshwater diatom biogeography and the genus Luticola: an extreme case of endemism in Antarctica. Polar Biol. 40, 1185–1196. doi: 10.1007/s00300-017-2090-7

[ref24] Kociolek J. P. Spaulding S. A. (2003). “Eutrophic and polluted waters” in Freshwater algae of North America. eds. Wehr J. D. Sheath R. G. (San Diego, CA: Academic Press), 637–653.

[ref25] Kohler T. J. Chatfield E. Gooseff M. N. Barrett J. E. McKnight D. M. (2015a). Recovery of Antarctic stream epilithon from simulated scouring events. Antarct. Sci. 27, 341–354. doi: 10.1017/S0954102015000024

[ref26] Kohler T. J. Fodelianakis S. Michoud G. Ezzat L. Bourquin M. Peter H.. (2022). Glacier shrinkage will accelerate downstream decomposition of organic matter and alters microbiome structure and function. Glob. Change Biol. 28, 3846–3859. doi: 10.1111/gcb.16169 PMC9323552 35320603

[ref27] Kohler T. J. Kopalová K. Van De Vijver B. Kociolek J. P. (2015b). The genus *Luticola* DG Mann (Bacillariophyta) from the McMurdo Sound region, Antarctica, with the description of four new species. Phytotaxa 208, 103–134. doi: 10.11646/phytotaxa.208.2.1

[ref28] Kohler T. J. Singley J. G. Wlostowski A. N. McKnight D. M. (2023). Nitrogen fixation facilitates stream microbial mat biomass across the McMurdo dry valleys, Antarctica. Biogeochemistry 166, 247–268. doi: 10.1007/s10533-023-01069-0

[ref29] Kohler T. J. Stanish L. F. Crisp S. W. Koch J. C. Liptzin D. Baeseman J. L.. (2015c). Life in the main channel: long-term hydrologic control of microbial mat abundance in McMurdo Dry Valley streams, Antarctica. Ecosystems 18, 310–327. doi: 10.1007/s10021-014-9829-6

[ref30] Kohler T. J. Stanish L. F. Liptzin D. Barrett J. E. McKnight D. M. (2018). Catch and release: Hyporheic retention and mineralization of N-fixing *Nostoc* sustains downstream microbial mat biomass in two polar desert streams. Limnol. Oceanogr. Lett. 3, 357–364. doi: 10.1002/lol2.10087

[ref31] Kohler T. J. Van Horn D. J. Darling J. P. Takacs-Vesbach C. D. McKnight D. M. (2016). Nutrient treatments alter microbial mat colonization in two glacial meltwater streams from the McMurdo dry valleys, Antarctica. FEMS Microbiol. Ecol. 92:fiw04. doi: 10.1093/femsec/fiw049 26940086

[ref32] Leibold M. A. Holyoak M. Mouquet N. Amarasekare P. Chase J. M. Hoopes M. F.. (2004). The metacommunity concept: a framework for multi-scale community ecology. Ecol. Lett. 7, 601–613. doi: 10.1111/j.1461-0248.2004.00608.x

[ref33] Maurice P. A. McKnight D. M. Leff L. Fulghum J. E. Gooseff M. (2002). Direct observations of aluminosilicate weathering in the hyporheic zone of an Antarctic Dry Valley stream. Geochim. Cosmochim. Acta 66, 1335–1347. doi: 10.1016/S0016-7037(01)00890-0

[ref34] McKnight D. M. Alger A. Tate C. Shupe G. Spaulding S. (1998). “Longitudinal patterns in algal abundance and species distribution in meltwater streams in Taylor valley, southern Victoria land, Antarctica” in Ecosystem dynamics in a Polar Desert: the McMurdo dry valleys, Antarctica. ed. Priscu J. C. (Washington, DC: American Geophysical Union), 109–127.

[ref35] McKnight D. M. Niyogi D. K. Alger A. S. Bomblies A. Conovitz P. A. Tate C. M. (1999). Dry valley streams in Antarctica: ecosystems waiting for water. Bioscience 49, 985–996. doi: 10.1525/bisi.1999.49.12.985

[ref36] McKnight D. M. Runkel R. L. Tate C. M. Duff J. H. Moorhead D. L. (2004). Inorganic nitrogen and phosphorous dynamics of Antarctic glacial meltwater streams as controlled by hyporheic exchange and benthic autotrophic communities. J. N. Am. Benthol. Soc. 23, 171–188. doi: 10.1899/0887-3593(2004)023<0171:INAPDO>2.0.CO;2

[ref37] McKnight D. M. Tate C. M. Andrews E. D. Niyogi D. K. Cozzetto K. Welch K.. (2007). Reactivation of a cryptobiotic stream ecosystem in the McMurdo dry valleys, Antarctica: a long-term geomorphological experiment. Geomorphology 89, 186–204. doi: 10.1016/j.geomorph.2006.07.025

[ref38] Muller K. (1974). Stream drift as a chronobiological phenomenon in running water ecosystems. Annu. Rev. Ecol. Syst. 5, 309–323. doi: 10.1146/annurev.es.05.110174.001521

[ref39] Müller-Haeckel A. (1971). Circadian periodicity of the colonization activity of drifting algae. Naturwissenschaften 58:273. doi: 10.1007/bf00603004 5580891

[ref40] Müller-Haeckel A. (1973). Different patterns of synchronization in diurnal and nocturnal drifting algae in the subarctic summer. Aquilo Ser. Zool. 14, 19–22,

[ref41] Oksanen J. Blanchet F. G. Michael F. Kindt R. Legendre P. McGlinn D. (2018). Vegan: community ecology package. R package. Available at: https://CRAN.R-project.org/package=vegan

[ref42] Peterson C. G. (1996). Mechanisms of lotic microalgal colonization following space-clearing disturbances acting at different spatial scales. Oikos 77, 417–435. doi: 10.2307/3545932

[ref43] Pfister L. Wetzel C. E. Klaus J. Martínez-Carreras N. Antonelli M. Teuling A. J.. (2017). Terrestrial diatoms as tracers in catchment hydrology: a review. Wires. Water 4:e1241. doi: 10.1002/wat2.1241

[ref44] R Core Team (2022). R: A language and environment for statistical computing. Vienna, Austria: R Foundation for Statistical Computing.

[ref45] Šabacká M. Priscu J. C. Basagic H. J. Fountain A. G. Wall D. H. Virginia R. A.. (2012). Aeolian flux of biotic and abiotic material in Taylor Valley, Antarctica. Geomorphology 155, 102–111. doi: 10.1016/j.geomorph.2011.12.009

[ref46] Sakaeva A. Sokol E. R. Kohler T. J. Stanish L. F. Spaulding S. A. Howkins A.. (2016). Evidence for dispersal and habitat controls on pond diatom communities from the McMurdo Sound region of Antarctica. Polar Biol. 39, 2441–2456. doi: 10.1007/s00300-016-1901-6

[ref47] Schulte N. O. Khan A. L. Smith E. W. Zoumplis A. Kaul D. Allen A. E.. (2022). Blowin’ in the wind: dispersal, structure, and metacommunity dynamics of Aeolian diatoms in the McMurdo Sound region, Antarctica. J. Phycol. 58, 36–54. doi: 10.1111/jpy.13223 34817069

[ref48] Singley J. G. Gooseff M. N. McKnight D. M. Hinckley E. S. (2021). The role of hyporheic connectivity in determining nitrogen availability: insights from an intermittent Antarctic stream. J. Geophys. Res. Biogeosci. 126:e2021JG006309. doi: 10.1029/2021JG006309

[ref49] Singley J. G. Salvatore M. R. Gooseff M. N. McKnight D. M. Hinckley E. L. S. (2023). Differentiating physical and biological storage of N along an intermittent Antarctic stream corridor. Freshw. Sci. 42, 229–246. doi: 10.1086/725676

[ref50] Soininen J. Teittinen A. (2019). Fifteen important questions in the spatial ecology of diatoms. Freshw. Biol. 64, 2071–2083. doi: 10.1111/fwb.13384

[ref51] Sokol E. R. Barrett J. E. Kohler T. J. McKnight D. M. Salvatore M. R. Stanish L. F. (2020). Evaluating alternative metacommunity hypotheses for diatoms in the McMurdo dry valleys using simulations and remote sensing data. Front. Ecol. Evol. 8:295. doi: 10.3389/fevo.2020.521668

[ref52] Stanish L. F. Kohler T. J. Esposito R. M. Simmons B. L. Nielsen U. N. Wall D. H.. (2012). Extreme streams: flow intermittency as a control on diatom communities in meltwater streams in the McMurdo dry valleys, Antarctica. Can. J. Fish. Aquat. Sci. 69, 1405–1419. doi: 10.1139/f2012-022

[ref53] Stanish L. F. Nemergut D. R. McKnight D. M. (2011). Hydrologic processes influence diatom community composition in Dry Valley streams. J. N. Am. Benthol. Soc. 30, 1057–1073. doi: 10.1899/11-008.1

[ref54] Tank J. L. Rosi-Marshall E. J. Griffiths N. A. Entrekin S. A. Stephen M. L. (2010). A review of allochthonous organic matter dynamics and metabolism in streams. J. N. Am. Benthol. Soc. 29, 118–146. doi: 10.1899/08-170.1

[ref55] Treonis A. M. Wall D. H. Virginia R. A. (1999). Invertebrate biodiversity in Antarctic Dry Valley soils and sediments. Ecosystems 2, 482–492. doi: 10.1007/s100219900096

[ref56] Vannote R. L. Minshall G. W. Cummins K. W. Sedell J. R. Cushing C. E. (1980). The river continuum concept. Can. J. Fish. Aquat. Sci. 37, 130–137,

[ref57] Welch K. A. Lyons W. B. Whisner C. Gardner C. B. Gooseff M. N. McKnight D. M.. (2010). Spatial variations in the geochemistry of glacial meltwater streams in the Taylor Valley, Antarctica. Antarct. Sci. 22, 662–672. doi: 10.1017/S0954102010000702

[ref58] Welschmeyer N. A. (1994). Fluorometric analysis of chlorophyll a in the presence of chlorophyll b and pheopigments. Limnol. Oceanogr. 39, 1985–1992. doi: 10.4319/lo.1994.39.8.1985

