# Supplementary material for: Drifting along: using diatoms to track the contribution of microbial mats to particulate organic matter transport in a glacial meltwater stream in the McMurdo Dry Valleys, Antarctica
Source: Front Microbiol. 2024 May 9;15:1352666. doi: 10.3389/fmicb.2024.1352666 (PMC11112031; doi:10.3389/fmicb.2024.1352666)
Supplement: Supplementary file 1 [file Data_Sheet_1.PDF]

Supplementary Material for

Drifting along: Using diatoms to track the contribution of microbial mats to particulate organic matter transport in a glacial meltwater stream in the McMurdo Dry Valleys, Antarctica

Lee F. Stanish[1], Tyler J. Kohler[1,2], Joshua Darling[1], Diane M. McKnight[1,3]

Affiliations
[1] Institute of Arctic and Alpine Research, University of Colorado, Boulder, Colorado, United States
[2] Department of Ecology, Faculty of Science, Charles University, Prague, Czechia
[3] Department of Civil and Environmental Engineering, University of Colorado, Boulder, Colorado, United States

Contents

Supplementary Figure 1
Supplementary Figure 2
Supplementary Figure 3
Supplementary Figure 4
Supplementary Figure 5
Supplementary Figure 6
Supplementary Figure 7
Supplementary Table 1
Supplementary Table 2

1.1 Supplementary Figures

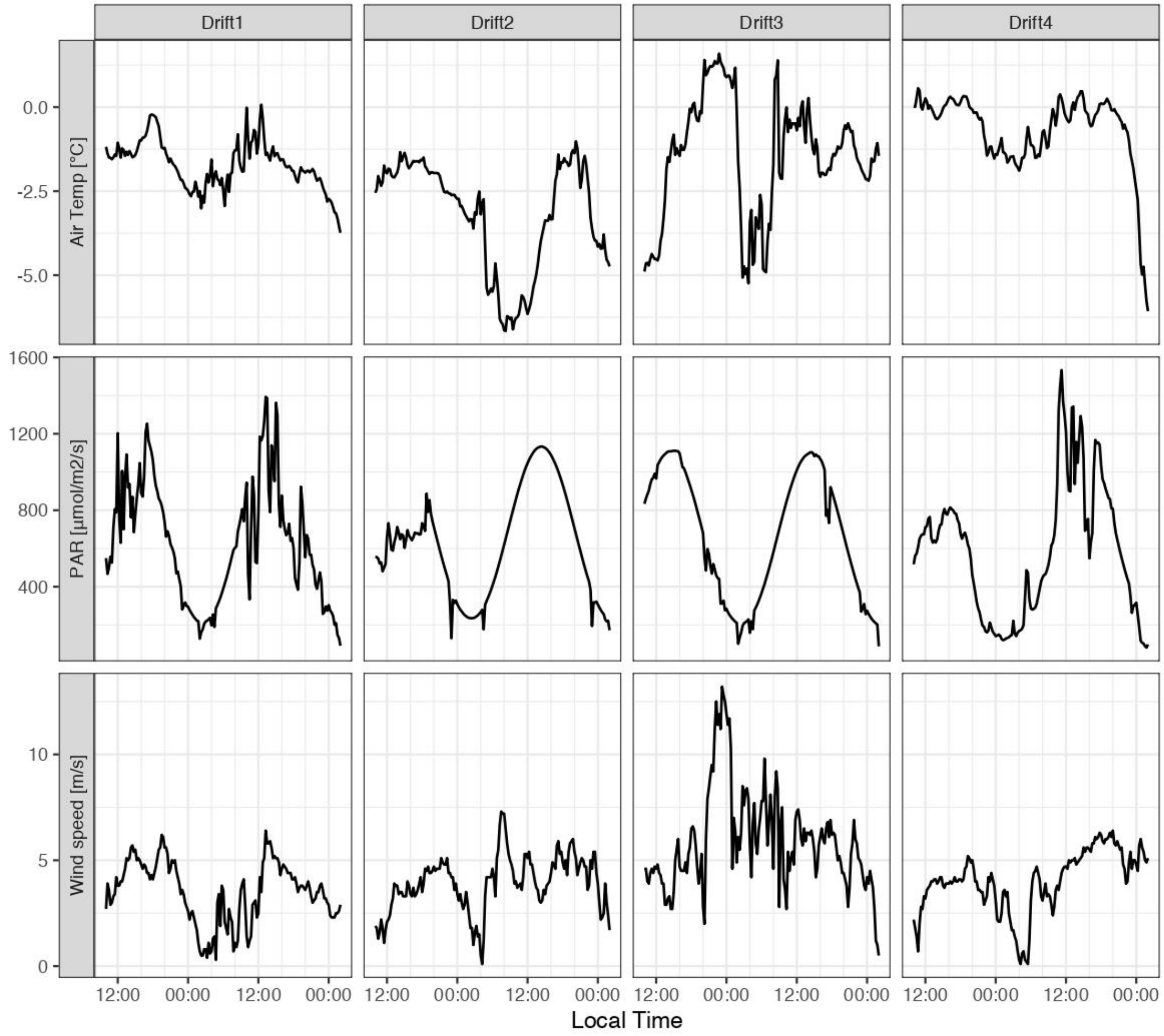

Supplemental Figure 1. Meteorological data over the course of each of the Drift experiments (1-4, as columns), including air temperature (top row), photosynthetically active radiation (middle row), and wind speed (bottom row). Data were taken from a meteorological station situated nearby the Von Guerard Stream gauge.

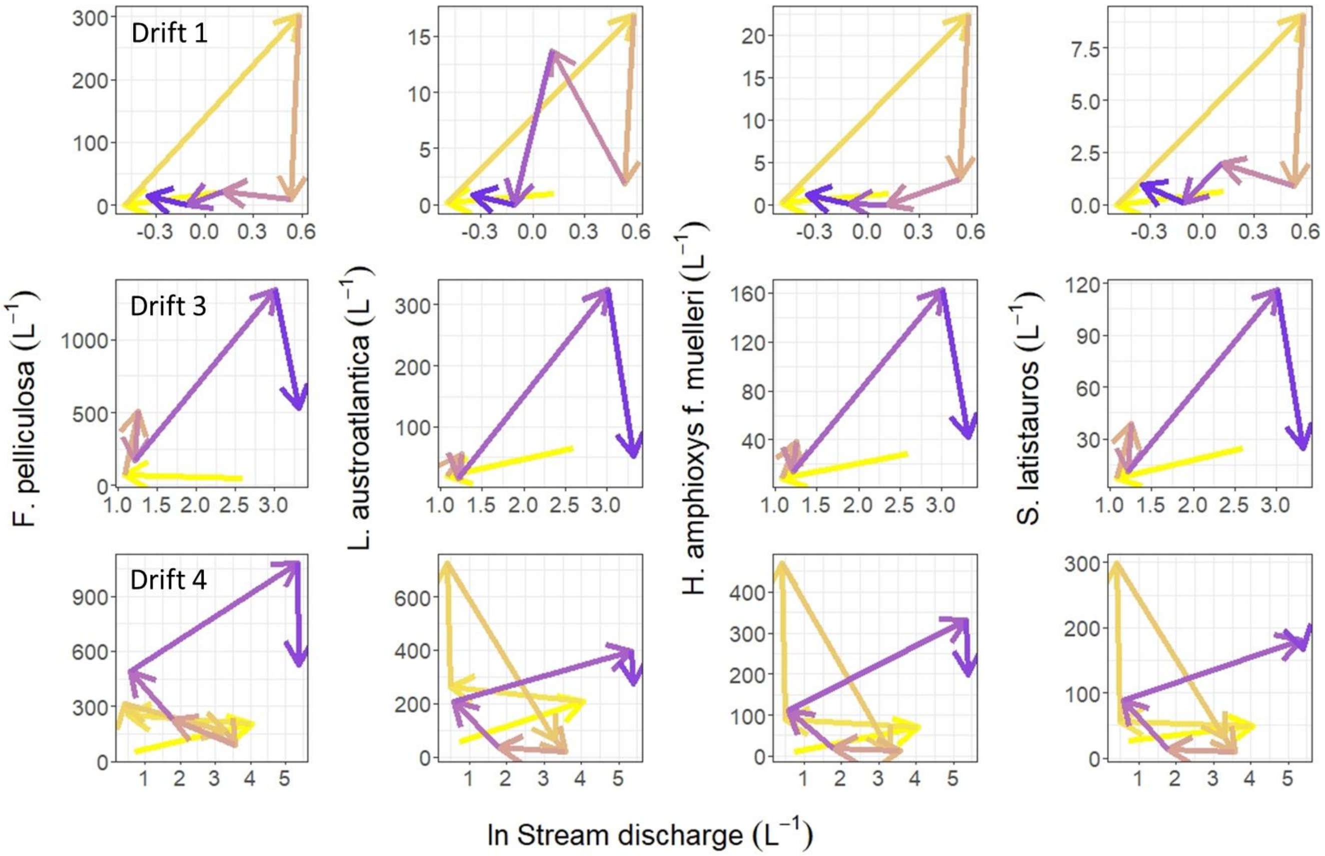

Supplemental Figure 2. Hysteresis in the relationship between Von Guerard Stream discharge (natural log transformed) and concentrations of four of the most common diatom species identified from the Drift experiments (from left to right, *Fistulifera pelliculosa*, *Luticola austroatlantica*, *Hantzschia amphioxys* f. *muelleri*, and *Stauroneis latistauros*). The Drift 1, 3, and 4 experiments are given as rows, with the four species as columns. Arrows indicate the clockwise relationship between discharge and concentration over the daily flow cycle. Please note difference in y-axes between panels.

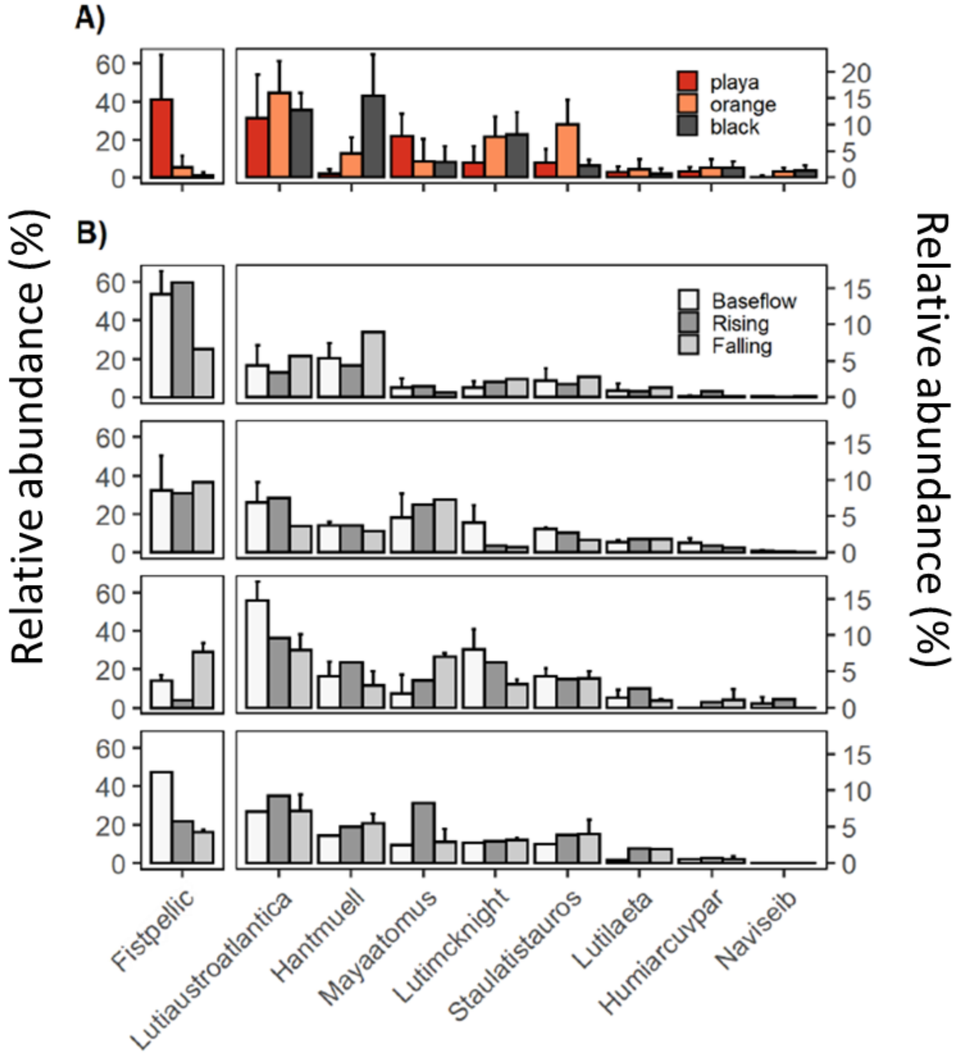

Supplemental Figure 3. Mean relative abundances (±SD) of the nine most abundant diatoms in the POM, orange channel mat, black marginal mat, and playa sediments samples. In A) samples are separated by habitat type, while in B) samples are separated by Drift experiment (1-4, top to bottom) and bars are colored by the portion of the hydrograph they represent (baseflow, rising limb, falling limb). For all, the small and lightly-silicified *Fistulifera pelliculosa* (Fistpellic) is plotted on a different y-axis due to its dominance in samples. For all other taxa, abbreviations are as follows: Lutiaustroatlantica = *Luticola austroatlantica*, Hantmuell = *Hantzschia amphioxys* f. *muelleri*, Mayaatomus = *Mayamaea atomus*, Lutimcknight = *Luticola macknightiae*, Staulatistauros = *Stauroneis latistauros*, Lutilaeta = *Luticola laeta*, Humiarcuvpar = *Humidophila arcuata* var. *parallela*, Naviseib = *Navicula seibigiana*.

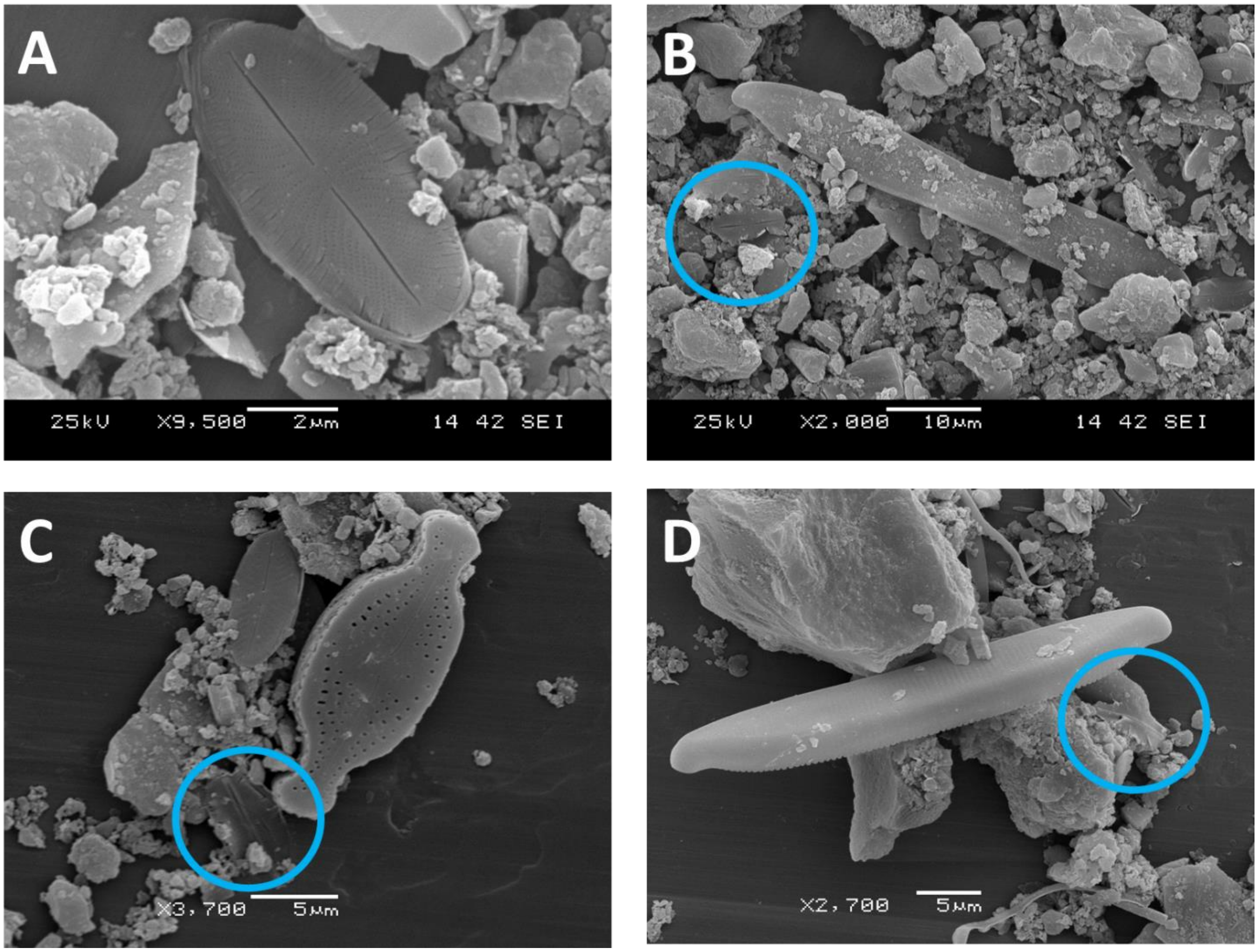

Supplemental Figure 4. Plate highlighting the unique characteristics of *Fistulifera pelliculosa* (i.e. light silicification, fine features, small size) with scanning electron micrographs from microbial mats taken from Wales Stream, another stream from Taylor Valley. Panel A shows a detail of the *F. pelliculosa* valve face. Remaining panels show *F. pelliculosa* (highlighted with blue circles) in relationship to B) *Nitzschia australocommutata*, C) *Luticola dolia* (above-right relative to *F. pelliculosa*) and *Mayamaea sp.* (directly above the *F. pelliculosa*), and D) a *Hantzschia sp*. Notice the difference in scale bars on panels. Many thanks to K. Kopalová for assisting with images.

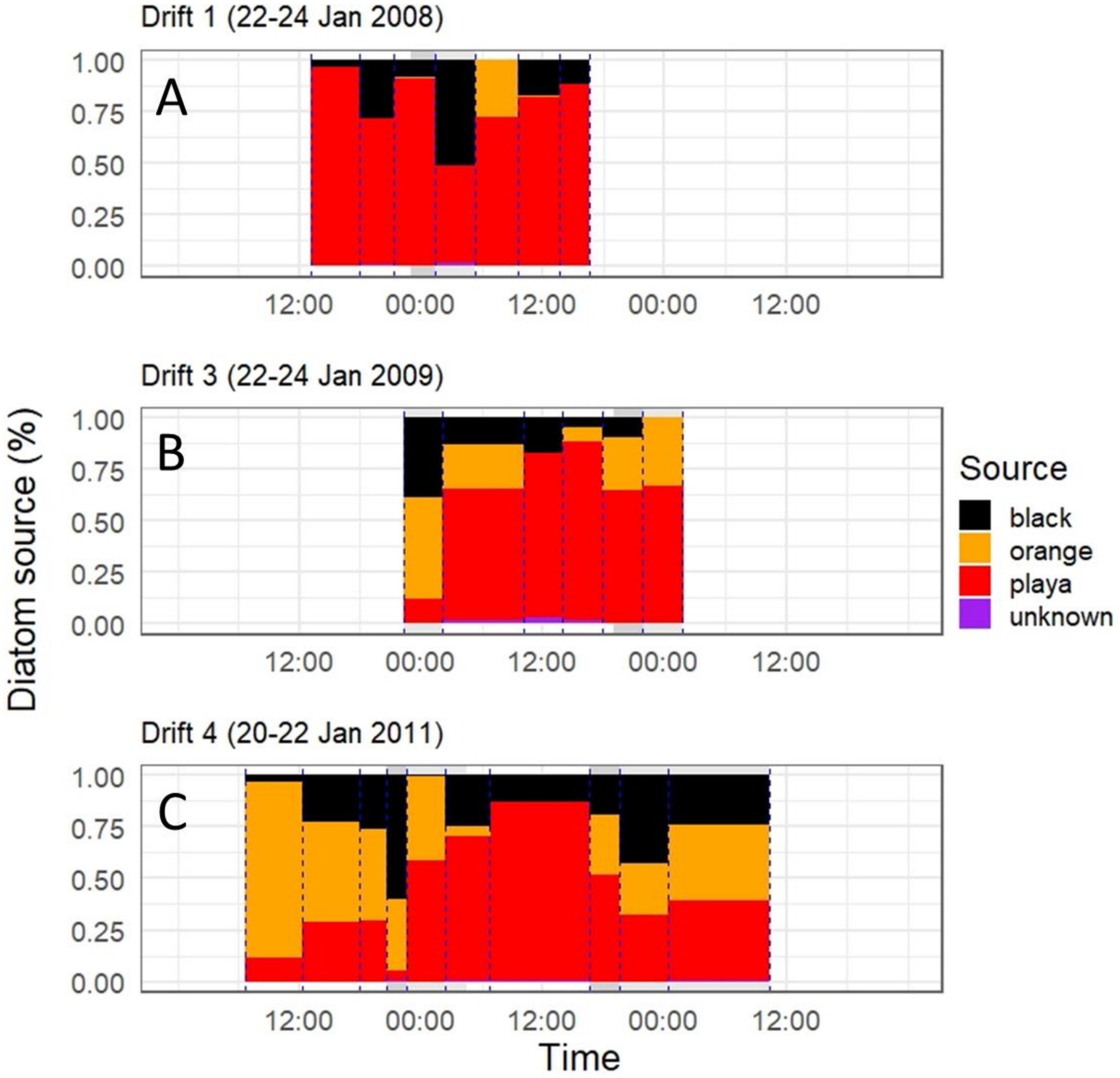

Supplemental Figure 5. The percent contribution of different diatom sources to the total POM diatom assemblage over the three Drift experiments as identified by the SourceTracker program. Bar width corresponds to the collection period of nets within Von Guerard Stream, and each vertical blue line corresponds with a time the collection net went in/out of the stream. The shaded area of each bar corresponds to the contribution of black mats (black), orange mats (orange), bare 'playa' sediments (red), and unknown sources (purple). Dark grey shaded areas in background indicate the rising limb of the hydrograph, and light grey bars indicate the falling limb, while other areas indicate baseflow conditions. Please note difference in y-axes between panels.

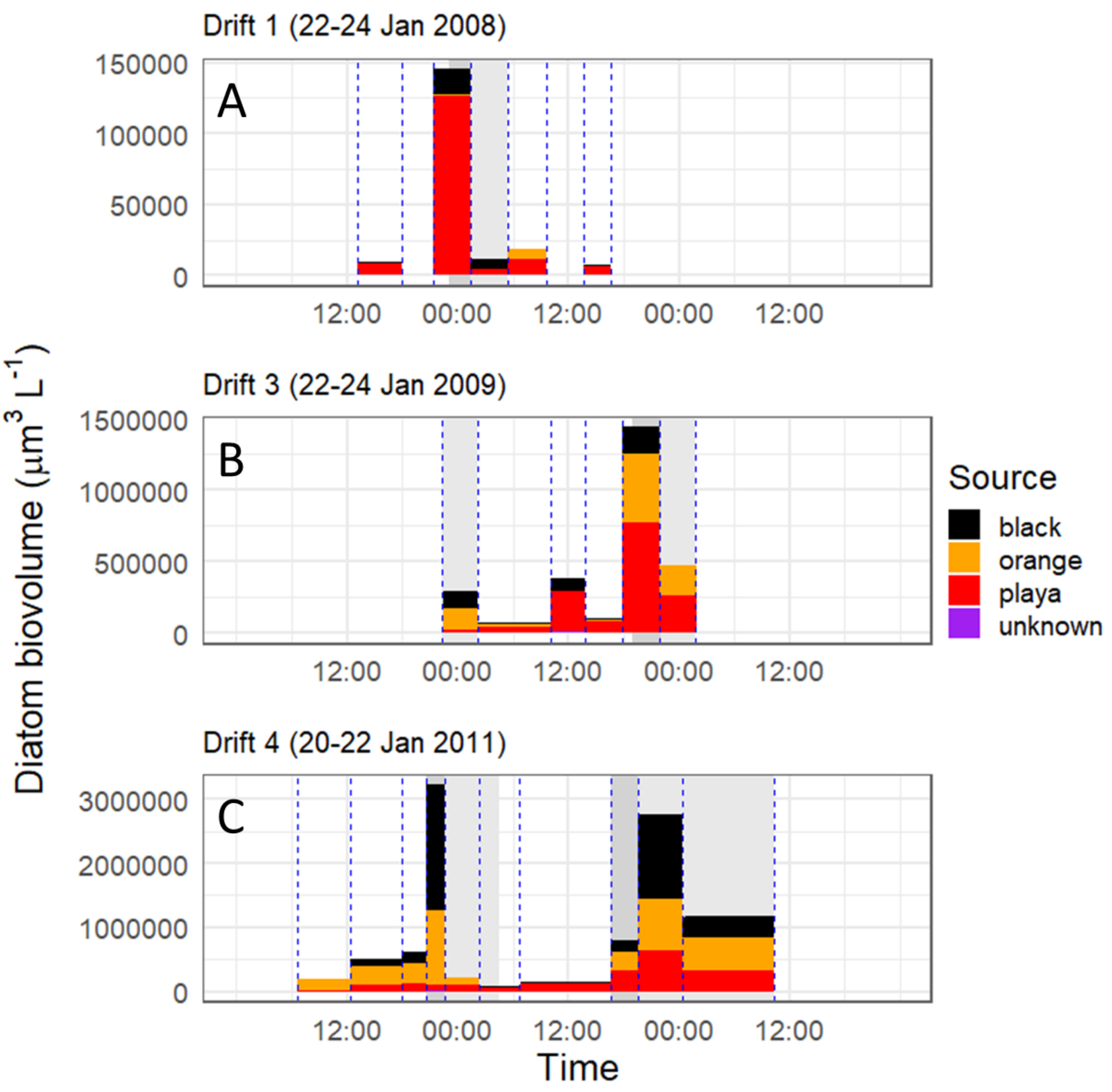

Supplemental Figure 6. Diatom biovolume concentrations over the three Drift experiments. Here, the percent modeled contribution for each habitat type is normalized by the diatom biovolume concentration at that timepoint. Bar width corresponds to the collection period of nets within Von Guerard Stream, and each vertical blue line corresponds with a time the collection net went in/out of the stream. The shaded area of each bar corresponds to the contribution of black mats (black), orange mats (orange), bare 'playa' sediments (red), and unknown sources (purple). Dark grey shaded areas in background indicate the rising limb of the hydrograph, and light grey bars indicate the falling limb, while other areas indicate baseflow conditions. Please note difference in y-axes between panels.

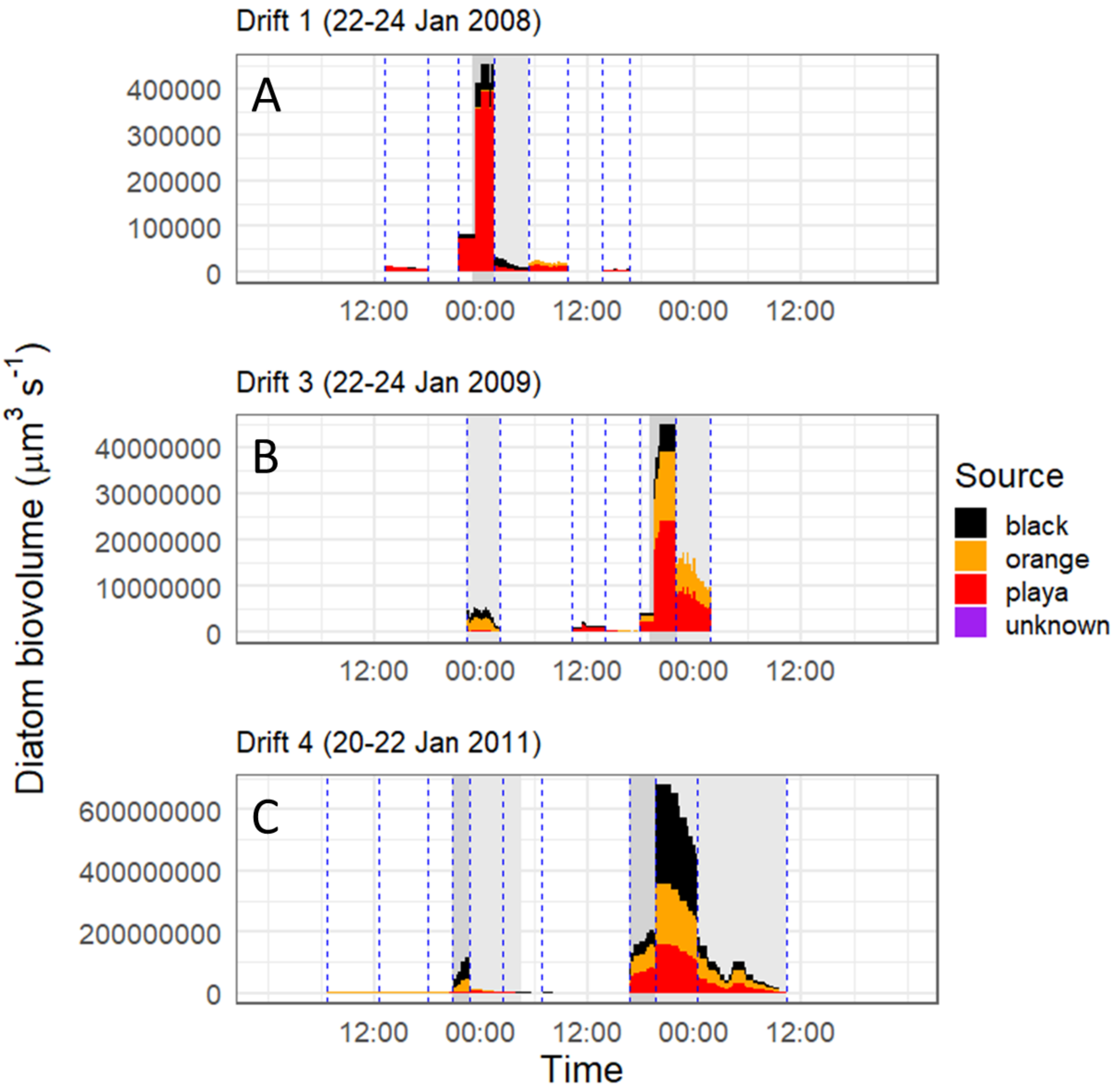

Supplemental Figure 7. Flux of diatom biovolume over the three Drift experiments. Here, diatom biovolume concentrations are corrected by discharge measured at 15 minute intervals at the Von Guerard Stream gauge. Vertical blue lines correspond with a time the collection net went in/out of the stream. Shaded areas correspond to the contribution of black mats (black), orange mats (orange), bare ‘playa’ sediments (red), and unknown sources (purple). Dark grey shaded areas in background indicate the rising limb of the hydrograph, and light grey bars indicate the falling limb, while other areas indicate baseflow conditions. Please note difference in y-axes between panels.

.

1.2 Supplementary Tables

Supplemental Table 1. List of samples from Von Guerard Stream used for diatom community analysis. Accession numbers are from the Antarctic Diatoms Database.

Accession No.	Sample ID	Season	Sample Type	Habitat
444	VGLO94S2	1993-94	orange mat	channel
443	VGLO94S1	1993-94	orange mat	channel
224	VGLB94S5	1993-94	black mat	margin
260	VGLB94S3	1993-94	black mat	margin
505	VGLO03S3	2002-03	orange mat	channel
729	VGLB03S3	2002-03	black mat	margin
727	VGLB03S1	2002-03	black mat	margin
710	VGLO07S4	2006-07	orange mat	channel
709	VGLO07S3	2006-07	orange mat	channel
708	VGLO07S2	2006-07	orange mat	channel
706	VGLB07S3	2006-07	black mat	margin
705	VGLB07S2	2006-07	black mat	margin
704	VGLB07S1	2006-07	black mat	margin
717	VGLO07.8S3	2007-08	orange mat	channel
718	VGLO_07.8Sb	2007-08	orange mat	channel
713	VGLB07.8S3	2007-08	black mat	margin
711	VGLB07.8S1	2007-08	black mat	margin
719	D1S1	2007-08	Drift 1 POM	water
720	D1S2	2007-08	Drift 1 POM	water
721	D1S3	2007-08	Drift 1 POM	water
722	D1S4	2007-08	Drift 1 POM	water
723	D1S5	2007-08	Drift 1 POM	water
724	D1S6	2007-08	Drift 1 POM	water

726	D1S7	2007-08	Drift 1 POM	water
889	D2S1	2008-09	Drift 2 POM	water
890	D3S1	2008-09	Drift 3 POM	water
891	D3S2	2008-09	Drift 3 POM	water
892	D3S3	2008-09	Drift 3 POM	water
893	D3S4	2008-09	Drift 3 POM	water
894	D3S5	2008-09	Drift 3 POM	water
895	D3S6	2008-09	Drift 3 POM	water
1137	VGLO10S2	2009-10	orange mat	channel
1138	VGLO10S3	2009-10	orange mat	channel
1139	VGLO10S4	2009-10	orange mat	channel
1433	drift4c	2010-11	Drift 4 POM	water
1436	drift4d	2010-11	Drift 4 POM	water
1439	drift4e	2010-11	Drift 4 POM	water
1443	drift4f	2010-11	Drift 4 POM	water
1448	drift4g	2010-11	Drift 4 POM	water
1453	drift4h	2010-11	Drift 4 POM	water
1458	drift4i	2010-11	Drift 4 POM	water
1463	drift4j	2010-11	Drift 4 POM	water
1468	drift4k	2010-11	Drift 4 POM	water
1473	drift4l	2010-11	Drift 4 POM	water
2413	PlayaS1	2012-13	playa sediments	channel
2417	PlayaS3	2012-13	playa sediments	channel
2421	PlayaS5	2012-13	playa sediments	channel
3659	playa2_19.20	2018-19	playa sediments	channel
3658	playa3_19.20	2018-19	playa sediments	channel
3660	playa4_19.20	2018-19	playa sediments	channel
3657	playa5_19.20	2018-19	playa sediments	channel

Supplemental Table 2. Results from the indicator species analysis. Below are listed the significant taxa from each habitat type along with the associated test statistic and p-value. There were no significant indicator taxa for the POM samples.

	Taxon	Statistic	p-value
**Black mats (11 species)**	*Hantzschia amphioxys* f. *muelleri*	0.785	0.005
	Hantzschia spp.	0.767	0.005
	Navicula seibigiana	0.695	0.005
	Humidophila arcuata	0.671	0.030
	Achnanthes taylorensis	0.666	0.015
	Muelleria spp.	0.632	0.010
	Chamaepinnularia cymatopleura	0.627	0.040
	Muelleria meridionalis	0.623	0.050
	Humidophila australis	0.617	0.015
	Luticola spp.	0.608	0.030
	Psammothidium germainii	0.471	0.050
**Orange mats (4 species)**	*Stuaroneis latistauros*	0.729	0.005
	Craticula molestiformis	0.728	0.005
	Luticola laeta	0.610	0.015
	Luticola austroatlantica	0.600	0.015
**Playa sediment (4 species)**	*Amphora oligotrophenta*	0.751	0.005
	Fistulifera pelliculosa	0.722	0.005
	Mayamaea atomus	0.650	0.005
	Mayamaea atomus v. *permitis*	0.554	0.045
